# Concomitant inhibition of PI3K/mTOR signaling pathways boosts antiproliferative effects of lanreotide in bronchopulmonary neuroendocrine tumor cells

**DOI:** 10.3389/fphar.2024.1308686

**Published:** 2024-02-05

**Authors:** Claus von Hessert-Vaudoncourt, Sara Lelek, Christina Geisler, Teresa Hartung, Vanessa Bröker, Franziska Briest, Liliana Mochmann, Fabian Jost-Brinkmann, Dagmar Sedding, Joana Benecke, Helma Freitag, Sebastian Wolfshöfer, Hedwig Lammert, Svenja Nölting, Michael Hummel, Jörg Schrader, Patricia Grabowski

**Affiliations:** ^1^ Medical Clinic III, Hematology, Oncology, Tumor Immunology, Charité-Universitätsmedizin Berlin, Berlin, Germany; ^2^ Department of Hepatology and Gastroenterology, Charité-Universitätsmedizin Berlin, Corporate Member of Freie Universität Berlin and Humboldt-Universität zu Berlin, Berlin, Germany; ^3^ Institute of Medical Immunology, Charité-Universitätsmedizin Berlin, Berlin, Germany; ^4^ Institute of Pathology, Charité-Universitätsmedizin Berlin, Berlin, Germany; ^5^ Department of Endocrinology, Diabetology and Clinical Nutrition, Universitätsspital Zürich, Zurich, Germany; ^6^ Department of Internal Medicine II, Klinikum der Universität München, Ludwig-Maximilians-Universität München, Munich, Germany; ^7^ I. Department of Medicine, Universitätsklinikum Hamburg-Eppendorf, Hamburg, Germany

**Keywords:** somatostatin analogues, lanreotide, BYL719, alpelisib, everolimus, RAD-001, bronchopulmonary neuroendocrine tumor, lung neuroendocrine tumor

## Abstract

**Introduction:** Somatostatin analogues (SSAs) are commonly used in the treatment of hormone hypersecretion in neuroendocrine tumors (NETs), however the extent to which they inhibit proliferation is much discussed.

**Objective:** We studied the antiproliferative effects of novel SSA lanreotide in bronchopulmonary NETs (BP-NETs). We focused on assessing whether pretreating cells with inhibitors for phosphatidylinositol 3-kinase (PI3K) and mammalian target for rapamycin (mTOR) could enhance the antiproliferative effects of lanreotide.

**Methods:** BP-NET cell lines NCI-H720 and NCI-H727 were treated with PI3K inhibitor BYL719 (alpelisib), mTOR inhibitor everolimus and SSA lanreotide to determine the effect on NET differentiation markers, cell survival, proliferation and alterations in cancer-associated pathways. NT-3 cells, previously reported to express somatostatin receptors (SSTRs) natively, were used as control for SSTR expression.

**Results:** SSTR2 was upregulated in NCI-H720 and NT-3 cells upon treatment with BYL719. Additionally, combination treatment consisting of BYL719 and everolimus plus lanreotide tested in NCI-H720 and NCI-H727 led to diminished cell proliferation in a dose-dependent manner. Production of proteins activating cell death mechanisms was also induced. Notably, a multiplexed gene expression analysis performed on NCI-H720 revealed that BYL719 plus lanreotide had a stronger effect on the downregulation of mitogens than lanreotide alone.

**Discussion/Conclusion:** We report a widespread analysis of changes in BP-NET cell lines at the genetic/protein expression level in response to combination of lanreotide with pretreatment consisting of BYL719 and everolimus. Interestingly, SSTR expression reinduction could be exploited in therapeutic and diagnostic applications. The overall results of this study support the evaluation of combination-based therapies using lanreotide in preclinical studies to further increase its antiproliferative effect and ultimately facilitate its use in high-grade tumors.

## Introduction

Bronchopulmonary neuroendocrine tumors (BP-NETs) are reported to represent 20%–30% of all NETs and account approximately for 1%–2% of lung cancers in adults. This low percentage means that most research effort has been placed upon other more common types of NETs and lung cancers, reason for which there is relatively limited knowledge on disease management for BP-NETs (Barbieri et al., 2014; Caplin et al., 2014; Cives and Strosberg, 2015; Lo Russo et al., 2016; Nölting et al., 2017; Lenotti et al., 2021). Of note, in 2015 the European Neuroendocrine Tumor Society (ENETS) published recommendations for best practices in the management of BP-NETs (Caplin et al., 2015). In 2018, the World Health Organization (WHO) distinguished between two types of BP-NETs according to their increasing aggressiveness: typical carcinoid and atypical carcinoid (Rindi et al., 2018).

Somatostatin (SST) is a peptide hormone which has exocrine, endocrine, paracrine and autocrine inhibitory effects. In addition, it is reported to regulate tumor cell growth via activation of somatostatin receptors (SSTRs), which modulate mitogen-activated protein kinase (MAPK) activity and phosphotyrosine phosphatases, such as SHP1, SHP2 and DEP-1 (Barbieri et al., 2014). NETs are known to overexpress SSTRs, which provides a molecular basis for diagnostics and therapeutic intervention (Cives and Strosberg, 2015). Nevertheless, the atypical BP-NET subtype displays a weaker SSTR expression, which could hinder somatostatin analogue therapy (Righi et al., 2010; Vesterinen et al., 2019).

Lanreotide, a commercially available somatostatin analogue (SSA), was granted FDA and EMA approval in 2014 and 2015, respectively, for gastroenteropancreatic NETs (GEP-NETs) and carcinoid syndrome, after a randomized, double-blind, phase 3 study (CLARINET, NCT02683941) revealed that treatment with lanreotide significantly prolonged progression-free survival vs. placebo in GEP-NETs (Caplin et al., 2014). Lanreotide binds primarily to SSTR2 and with lower affinity to SSTR5, allowing for activation of intracellular pathways which induce antisecretory and antiproliferative effects. However, thus far few data is available on the antiproliferative effects of lanreotide or other SSAs in BP-NETs (Barbieri et al., 2014). The SPINET trial (NCT02683941), a randomized, double-blind, phase 3 study evaluating the efficacy and safety of lanreotide vs. placebo in patients with typical carcinoid and atypical carcinoid BP-NETs, was prematurely terminated due to slow accrual. This trial, the largest prospective study to date with an SSA in SSTR-positive BP-NETs, concluded that lanreotide 120 mg could be an appropriate treatment option, especially for typical carcinoids (Reidy-Lagunes et al., 2021).

A breakthrough 2017 study on GEP-NET and BP-NET cells associated treatment with phosphatidylinositol 3-kinase (PI3K) inhibitor with significant SSTR2 upregulation (Nölting et al., 2017). The same observation was found in GEP-NET cells after combined PI3K and mammalian target of rapamycin (mTOR) inhibitor treatment (Nölting et al., 2017). Reportedly, this has the potential of sensitizing NET cells to therapy with SSAs. Here we investigated the antiproliferative effects of lanreotide on BP-NET cells after applying single and combined doses of PI3K and mTOR inhibitors: BYL719 (also called alpelisib) (Fritsch et al., 2014) and everolimus (earlier code name RAD-001) (Everolimus, 2014). We additionally looked into the alterations of signaling pathways that may modulate tumoricidal effects as a result of this novel combination strategy.

## Materials and methods

### Cell culture and drugs

BP-NET cell line of atypical carcinoid origin NCI-H720 (H720) (ATCC, United States) was grown in Dulbecco’s modified Eagle’s medium (DMEM) (GIBCO, Germany), supplemented with 4.5 g/L L-glutamine, heat-inactivated 5% (v/v) fetal calf serum (FCS), 100 U/ml penicillin and 100 μg/mL streptomycin, 0.005 mg/mL insulin, 0.01 mg/mL transferrin, 30 nM sodium selenite, 10 nM hydrocortisone, 10 nM beta-estradiol and 4.5 mM L-glutamine (for all supplements: GIBCO, Germany). BP-NET cell line of typical carcinoid origin NCI-H727 (H727) (ATCC, United States) was grown in Roswell Park Memorial Institute medium (RPMI 1640) (GIBCO, Germany), supplemented with heat-inactivated 10% (v/v) fetal calf serum (FCS), 100 U/ml penicillin and 100 μg/mL streptomycin (for all additions: GIBCO, Germany) and 2.5% (v/v) 4-(2-hydroxyethyl)-1-piperazineethanesulfonic acid (HEPES) buffer (Biochrom, Germany). Both purchased cell lines underwent authentication at the DSMZ in Braunschweig, Germany, in 2014, and their neuroendocrine features were confirmed by immunocytochemistry (markers chromogranin A, synaptophysin, cytokeratin, vimentin and syntaxin). NT-3 cells, a recently established pancreatic neuroendocrine (PNET) cell line, are reported to highly express SSTRs (Benten et al., 2018), and were therefore here chosen as control of SSTR expression. They were cultivated in RPMI medium supplemented with 10% FCS, penicillin/streptomycin, HEPES, epidermal growth factor (EGF) (20 ng/mL) and fibroblast growth factor 2 (FGF2) (10 ng/mL) (Peprotech, Germany).

Cell culture was performed in sterile conditions under a biosafety cabinet (NuAire, United States). All cell lines were *Mycoplasma*-free, incubated at 37°C in a humidified cell culture incubator with 5% CO_2_ and were regularly passaged when 80% confluency was reached, split 1:2 or 1:3.

The SSA lanreotide was kindly provided by Ipsen (France), the selective PI3Kα inhibitor BYL719 was kindly provided by Novartis (Switzerland) and the mTOR complex 1 (mTORC1) inhibitor everolimus was purchased from Selleckchem (Germany). Drug combinations were checked for efficiency, drug-drug interactions and burden on metabolism using the drug cocktail optimization tool SuperCYP (Preissner et al., 2012).

### Real time quantitative PCR of SSTR subtypes after BYL719 monotreatment

NT-3 and H727 cells were incubated with BYL719 using an effective dose (10 μM) close to the IC_50_ discovered in a previous study (Nölting et al., 2017) for 48 h. Total RNA was isolated from cell cultures using the NucleoSpin RNA/Protein kit (Macherey-Nagel, Germany) according to manufacturer’s instructions. Total RNA was reverse-transcribed using the High Capacity cDNA Reverse Transcription Kit (Thermo Fisher Scientific, Germany) and a T3 Thermocycler (Biometra, Germany). We used pre-validated TaqMan Primers for SSTR2, SSTR5 and GAPDH (Thermo Fisher Scientific, Germany) for real time quantitative PCR (qRT-PCR) in a Step One Plus Real-Time PCR System (Thermo Fisher Scientific, Germany). The assay ID of SSTR2 and SSTR5 primers ordered from Thermo Fisher Scientific were Hs00265624_s1 and Hs00990407_s1, respectively. qRT-PCR values were expressed as cycle threshold (Ct) values normalized to housekeeping gene GAPDH using the 2^−ΔΔCt^ method (Livak and Schmittgen, 2001). Data are presented as mean ± standard deviation (SD) of at least three independent experiments with three replicates per data point.

### Immunocytochemical staining of SSTR subtypes after monotreatment or co-treatment

H720 and H727 cells were seeded in 25 cm^2^ flasks and underwent monotreatment with 10 μM BYL719 (48 h) or both 1 μM BYL729 and 1 nM everolimus (24 h). Untreated cells were given vehicle 0.1% DMSO (Roth, Germany). The adherent H727 cells were harvested and seeded on 8-well Multitest glass slides (MP Biomedicals, Germany). The glass slides were then placed on culture dishes and incubated overnight to allow proper attachment of the cells. H720 cells were seeded on glass slides directly using a CytoSpin Cytocentrifuge (Thermo Fisher Scientific, Germany).

Glass slides containing H720 and H727 cells were treated with primary antibodies SSTR2 (SC-25676) and SSTR5 (SC-25679) (Santa Cruz, Germany), diluted 1:100. Secondary antibody Goat anti-Rabbit IgG, Alexa Fluor 488 (Invitogen, United States) diluted 1:500 was applied 24 h later. All slides were mounted with cover slips using Roti-Mount FluorCare DAPI (Carl Roth, Germany). Fluorescent images were obtained using a Nikon fluorescence microscope and the bundled NIS software (Nikon, Germany). Each experiment was executed 4 times independently.

### Quantification of immunocytochemical staining

The scoring of each immunocytochemistry experiment was determined using Remmele and Stegner’s immunoreactive score (IRS) method (Remmele and Stegner, 1987). The IRS was calculated by multiplying the staining intensity (0 = no staining, 1 = weak staining, 2 = moderate staining, 3 = strong staining) by the percentage of positively stained cells (0 = 0%, 1 = less than 10%, 2 = 10–50%, 3 = 51–80%, 4 = more than 80%). IRS results of 0–2 were negative, 3–4 were low, 5 - 6 were intermediate, 7–12 were high.

### Cell survival assay after lanreotide exposure with/without pretreatment

H720 and H727 cells were seeded in 75 cm^2^ flasks and underwent pretreatment with either: 1) simultaneous 24 h exposure to 1 μM BYL719 and 1 nM everolimus, 2) 24 h exposure to 1 nM everolimus, or 3) 24 h exposure to 10 μM BYL719. Next, cells were transferred to 96-well plates and left to grow overnight. Cells were then incubated with different concentrations (0.1–10,000 nM) of lanreotide in quintuplicate for 24 h. Control cells received vehicle 0.1% DMSO. Lanreotide had to be re-applied every 48 h due to its half-life activity. In total, cells were exposed to lanreotide for 120 h (this duration takes into account the comparably high doubling time of the cell lines and follows the one used in a previous study) (Nölting et al., 2017). The cell proliferation reagent WST-1 (Roche Diagnostics GmbH, Germany) was added according to manufacturer’s instructions after lanreotide incubation. Measurement of absorption was performed using a Tecan Sunrise microplate reader (Tecan Trading AG, Germany). Results were normalized to control. Each experiment was carried out 3 times independently.

Based on the viability data, we calculated dose-response curves with GraphPad Prism 6 (GraphPad Software, United States) and determined the IC_50_ (Sebaugh, 2011). Dose-response curves were fitted to the measured data using the method of least squares with variable slope; goodness of fit was quantified with *R* ^2^ and sums of squares.

### CFSE-based cell division assay after combination treatment

To track proliferation of cells during the combination treatment with defined IC_50_ doses of lanreotide, H720 and H727 cells were labeled using the CFSE Cell Division Assay Kit (Cayman Chemical, United States) following manufacturer’s instructions. 5-Carboxyfluorescein N-Succinimidyl ester (CFSE) is a green fluorescent dye taken up by live cells. Cell division results in halving of the fluorescent signal, therefore proliferating populations will present successively decreasing brightness with each generation. This allows to distinguish actively proliferating cells from cells that are not dividing (Lyons, 2000; Parish et al., 2009). Cells were labeled on day 0 and the assay ran for 72 h. During this time, a series of inhibitors were added. First, 1 μM BYL719 was added immediately after labeling. 24 h later, 1 nM everolimus was added. Finally, 24 h after this, lanreotide (IC_50_) was added. Four timepoints from the assay length were selected for CFSE signal measurement: at 0 h, 24 h, 48 h and 72 h. For each timepoint there was a growth control which only received vehicle 0.1% DMSO. Fluorescence signals were detected by flow cytometry using the flow cytometer BD FACSCalibur and attendant software CellQuest Pro, and analyzed by FlowJo 8.7 (BD Biosciences, United States). A sample of unlabeled, untreated cells was used to determine autofluorescence levels. Results were normalized to the growth control. Two independent experiments with two biological replicates per condition were performed.

### Western blotting and immunodetection after combination treatment

Following pre-incubation with 1 μM BYL719 and 1 nM everolimus (24 h), plus lanreotide (IC_50_) exposure (24 h), cells were lysed in NP-40 buffer (Thermo Fisher Scientific, United States). The content of total protein was assessed using the Bradford method (Bradford M. M, 1976). 20 μg protein was used per lane. SDS-PAGE and Western blotting were performed using a standard protocol. Reversible Ponceau S staining (Sigma Aldrich, United States) was applied to check the protein transfer into the membranes. Membranes were blocked in 5% milk/TBS-T (m/v) for 1 h and incubated with recommended dilutions of primary antibody overnight. The following primary antibodies were used: p-AKT (Ser473) (#4060), p-AKT (Thr308) (#13038), AKT (pan) (#2920), p-4EB-P1 (Ser65) (#9451), 4E-BP1 (#9644), p27 Kip1 (#3686), p-RB (Ser780) (#9307), RB (#9309), BAX (#2772), p-ERK1/2 (Thr202/Tyr204) (#4370), ERK1/2 (#4695), p-mTOR (#2971S), mTOR (#2972S), cleaved PARP (Asp214) (#5625), p-FOXO1 (Thr24) (#9464), FOXO1 (#2880P) (all from Cell Signaling, United States), IGF-1Rα (N-20) (sc-712), IGF-1Rβ (C-20) (sc-25676), SSTR2 (H-50) (sc-25676) and SSTR5 (H-54) (sc-25679) (all from Santa Cruz, United States). β-tubulin (T5201) (Sigma Aldrich, United States) was used as loading control.

After incubation with secondary HRP-labeled antibody (DAKO, Agilent, United States), we used the ECL Prime Western Blotting Reagent (Amersham, United Kingdom) with a LAS 4000 luminescent image analyzer (Fujifilm, Germany) for detection. For re-probing, antibody binding was removed with acidic glycine buffer (pH 2.3). Optical density of the bands was densitometrically quantified using ImageJ 1.440 software (National Institute of Health, United States). Band intensities were quantified from at least 3 independent experiments for each cell line and protein (except for mTOR, AKT (pan) and cleaved PARP with two independent experiments for each), normalized to the respective loading control and compared with the vehicle control.

### Multiplexed gene expression analysis of cancer-associated pathways (NanoString technology)

H720 cells were seeded in 75 cm^2^ flasks and underwent treatment with either only 10 μM BYL719 (48 h), or pretreatment with 10 μM BYL729 (48 h) prior to lanreotide (IC_50_) exposure (24 h). Control received vehicle 0.1% DMSO. Two biological replicates per condition were used.

RNA was extracted using the RNeasy Mini Kit (Qiagen, Germany), following manufacturer’s instructions. Total RNA content of each sample was assessed using the 2200 TapeStation system (Agilent, United States) and the Qubit Fluorometer (Thermo Fisher Scientific, Germany). 60 ng RNA per sample were used in the nCounter^®^ PanCancer Pathway Panel gene expression analysis (NanoString Technologies, United States). This novel technology contains 770 different probes whose sequences are complementary to corresponding target mRNAs (genes involved in 13 cancer-associated canonical pathways). Hybridization took place at 65°C overnight. The probes were labeled with gene-specific fluorescence bar codes and immobilized on a coated slide. Sample preparation was conducted with nCounter PrepStation 5 s, and microscopy scanning of the bar code signals with nCounter Digital Analyzer 5 s (NanoString Technologies, United States). The data were analyzed by first principal component analysis using nSolver 2.5 PanCancer Pathway Analysis Module software (NanoString Technologies, United States). The resulting absolute log fold change data on the single genes were summed up into pathway scores. We selected only genes with fold changes lower than 0.5 and greater than 1.5, combined with *p*-values lower than 0.05, to discover major differentially expressed genes. The Benjamini and Hochberg procedure was used to calculate the false discovery rate (FDR) (Benjamini and Hochberg, 1995).

### Statistical analysis

Graphs and statistical analyses were performed with Microsoft Excel (Microsoft, United States) and GraphPad Prism 6 (GraphPad Software, United States), with use of standard two-tailed unpaired Student’s t-test for normally distributed data. The data passed a Kolmogorov-Smirnov test for normal distribution, appropriate for data sets below 20 data points. All data were expressed as means of at least three independent experiments (±SD), unless otherwise stated. *p*-values lower than 0.05 were considered statistically significant.

## Results

### mRNA expression of NET markers SSTRs after PI3K inhibitor treatment: differential marker expression in NT-3 and H727 cells

**FIGURE 1:**
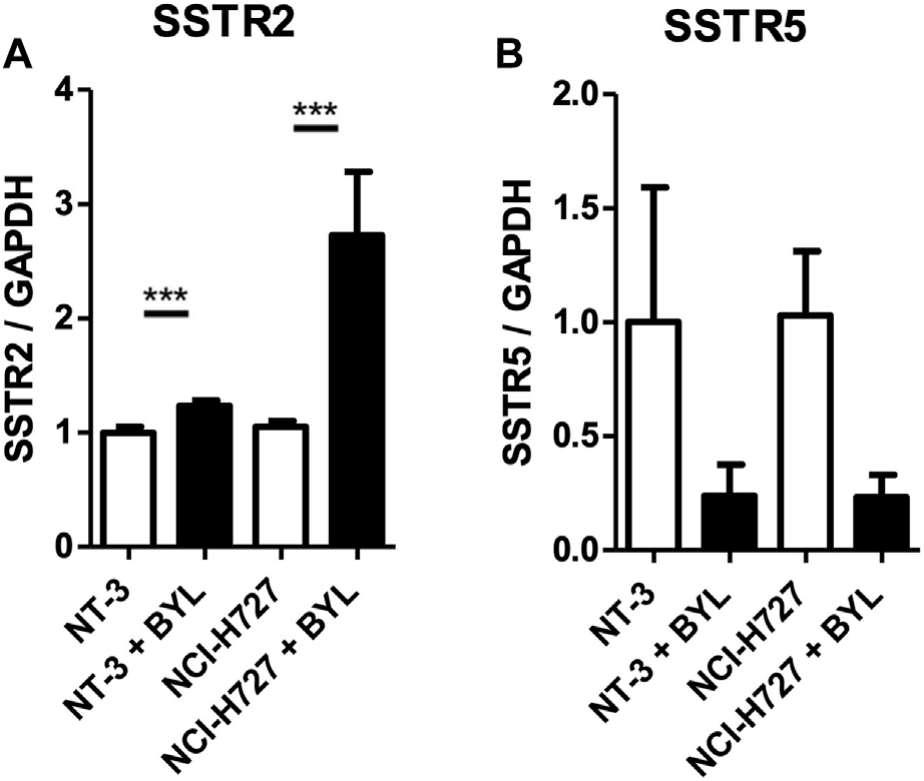
*In vitro* modulation of NET markers SSTR2 and SSTR5 in NT-3 and H727 cells after PI3K inhibitor treatment. NT-3 and H727 cells were incubated with 10 μM BYL719 for 48 h and mRNA expression of SSTR2 **(A)** and SSTR5 **(B)** was analyzed by qPCR. Values are summarized from three independent experiments of three replicates per data point. Marker expression was normalized to GAPDH and compared with vehicle control. *, **, ***: significant, very significant and extremely significant (*p* ≤ 0.05; *p* ≤ 0.01; *p* ≤ 0.001).

Treatment of recently established PNET cell line NT-3 and pulmonary typical carcinoid cell line H727 with 10 μM BYL719 for 48 h induced mRNA expression of SSTR2, but not SSTR5, two genes related to neuroendocrine differentiation. While SSTR2 induction in NT-3 cells was minimal, we observed a 2.5- (CI 1.8 ± 3.1) fold induction of SSTR2 mRNA in H727 cells. Interestingly, transcripts for SSTR5 decreased in both cell lines upon BYL719 treatment (*p* < 0.001) (shown in Figure 1).

### Detection of NET markers after combined PI3K/mTOR inhibitors treatment: induced expression of SSTR2 and SSTR5 in H720 cells, and SSTR2 in H727 cells

**FIGURE 2:**
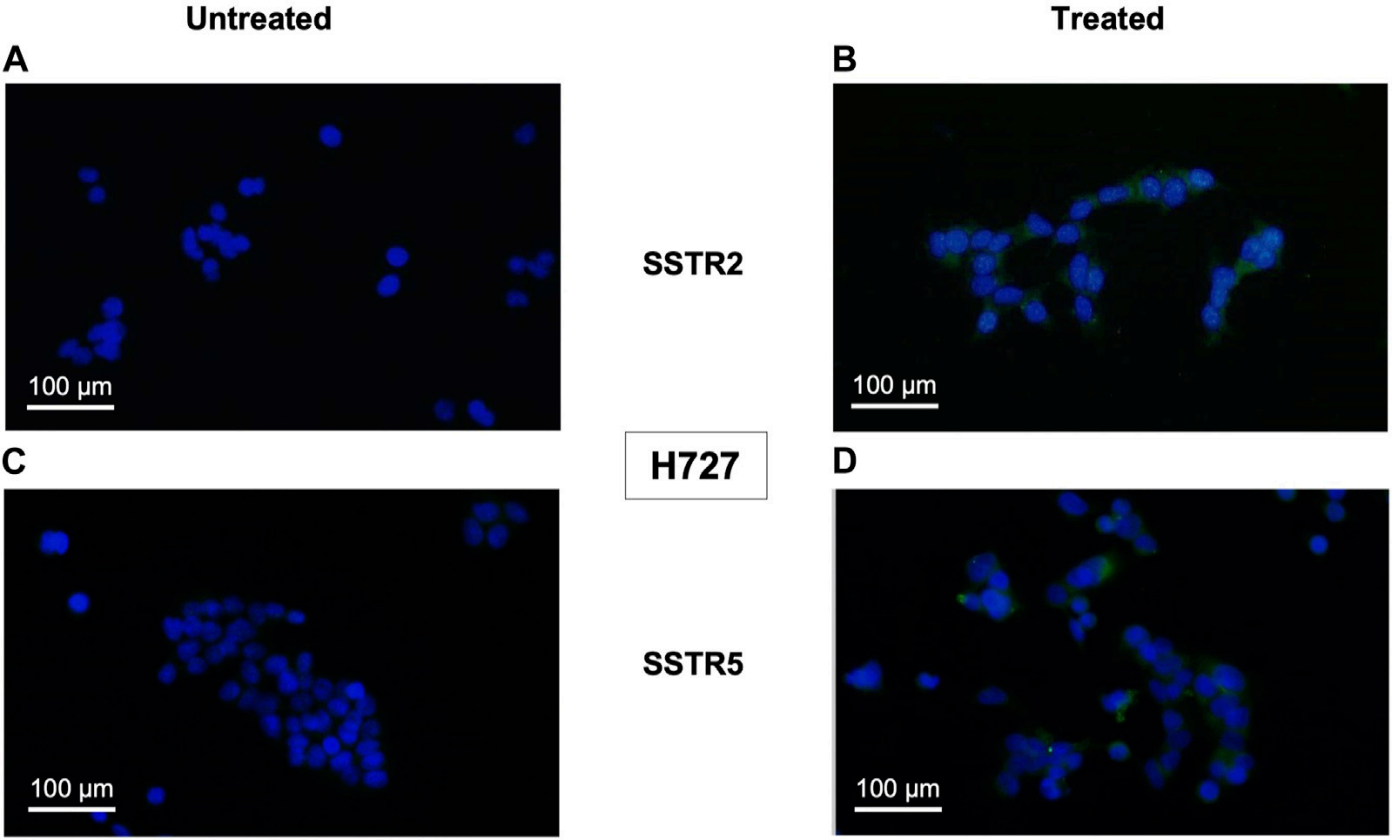
Immunocytochemical expression of SSTRs in monotreatment of H727 cells are displayed as representative examples (fluorescence microscopy, ×100 magnification). H727 cells were incubated with 10 μM BYL719 for 48 h and stained fort SSTRs. Nuclei were counterstained with DAPI. **(A,B)**: Composite images (DAPI and FITC channels) of untreated (left) and treated (right) H727 cells stained for SSTR2. **(C,D)**: Composite images (DAPI and FITC channels) of untreated (left) and treated (right) H727 cells stained for SSTR5.

**FIGURE 3:**
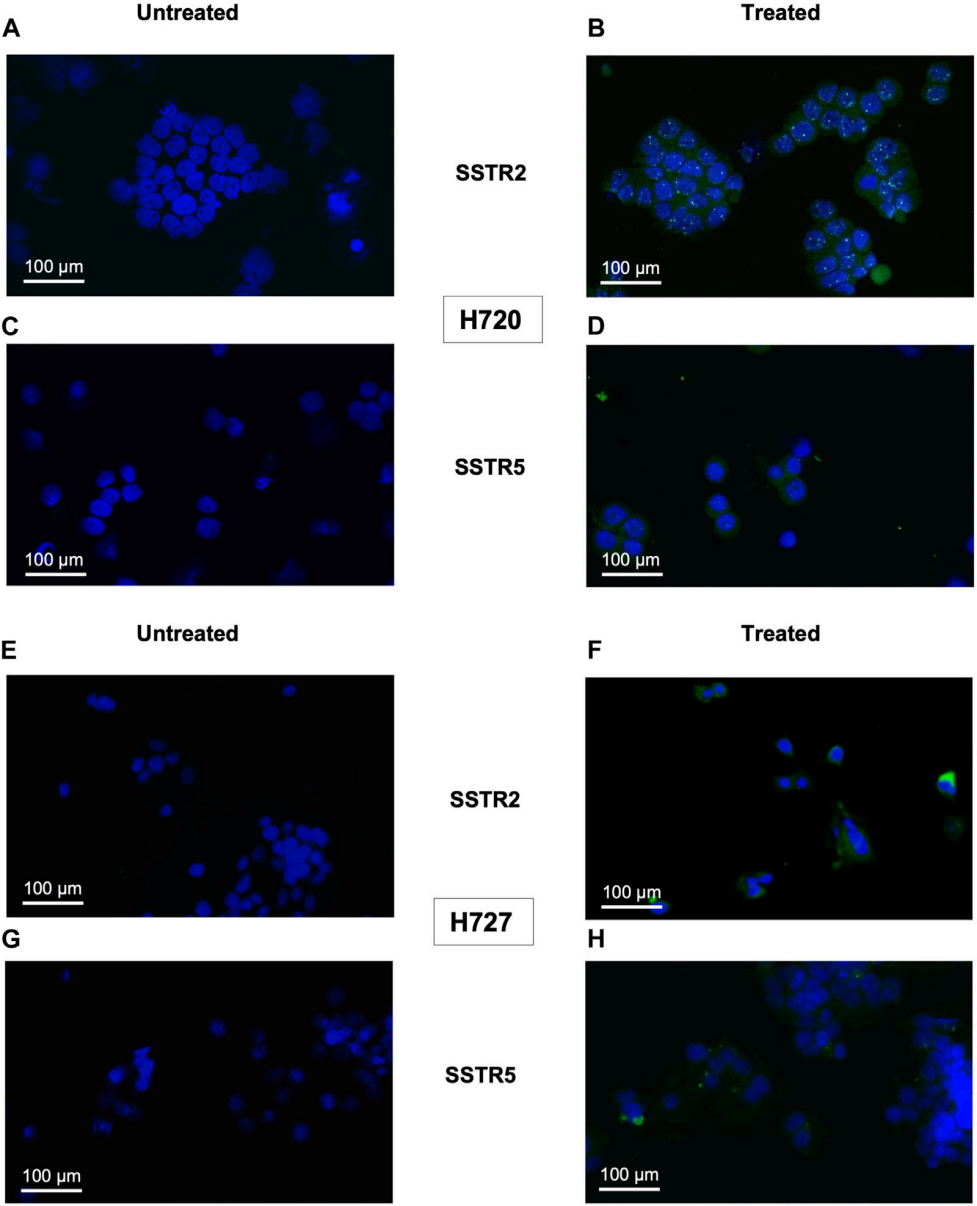
Immunocytochemical expression of SSTRs in co-treatment of H720 and H727 cells: representative examples (fluorescence microscopy, ×100 magnification). H720 and H727 cells were incubated with 1 μM BYL719 and 1 nm everolimus for 24 h and stained for SSTRs. Nuclei were counterstained with DAPI. **(A,B)**: Composite images (DAPI and FITC channels) of untreated (left) and treated (right) H720 cells stained for SSTR2. **(C,D)**: Composite images (DAPI and FITC channels) of untreated (left) and treated (right) H720 cells stained for SSTR5. **(E,F)**: Composite images (DAPI and FITC channels) of untreated (left) and treated (right) H727 cells stained against SSTR2. **(G,H)**: Composite images (DAPI and FITC channels) of untreated (left) and treated (right) H727 cells stained for SSTR5.

Monotreatment of cells with PI3K inhibitor BYL719 and co-treatment with BYL719 and mTOR inhibitor everolimus induced expression of proteins related to neuroendocrine differentiation in BP-NET cells. This was observed in the immunocytochemistry of SSTR2 and SSTR5, which was positive for this staining (shown in Figures 2, 3). The authors declare not having observed significant morphological changes as a result of these treatments.

Treatment with 10 μM BYL719 for 48 h was assayed in H727 cells and revealed induction of expression of receptors SSTR2 (*p* = 0.003) and SSTR5 (*p* = 0.001). Co-treatment with 1 μM BYL719 and 1 nM everolimus for 24 h was tested in both typical carcinoid H727 cells and atypical carcinoid H720 cells. It induced expression of SSTR2 (*p* = 0.0001) and SSTR5 (*p* = 0.002) in H720 cells, and expression of SSTR2 (*p* = 0.01) in H727 cells. The overall immunostaining pattern was SSTR2 > SSTR5, with all treated samples showing higher IRS values for SSTR2 compared to IRS values for SSTR5 (shown in Tables 1 and 2). High IRS values were seen for SSTR2 in six of the eight co-treated samples (75%), with moderate staining intensity in the remaining ones. The same eight co-treated samples had different SSTR5 staining intensities (low, moderate, and high). High SSTR5 staining intensity was most often seen in H720 cells. The combination 1 μM BYL719 plus 1 nM everolimus in H720 cells exerted an additive effect that produced a significant overexpression of SSTR2, compared to the expression observed with 10 μM BYL719 treatment (*p* = 0.03).

**TABLE 1:** Expression of SSTR2 and SSTR5 on H727 cells after monotreatment with BYL719, as determined by the immunoreactive score (IRS) according to Remmele and Stegner (Preissner et al., 2012), from four independent experiments. Negative (IRS = 0–2), low (IRS = 3–4), intermediate (IRS = 5–6), and high (IRS = 7–12).

NET marker IRS		Treated H727 (n = 4)
SSTR2	Negative	0 (0%)
	Low	1 (25%)
	Intermediate	2 (50%)
	High	1 (25%)
SSTR5	Negative	0 (0%)
	Low	3 (75%)
	Intermediate	1 (25%)
	High	0 (0%)

**TABLE 2:** Expression of SSTR2 and SSTR5 in H720 and H727 cells after co-treatment with BYL719 and everolimus, as determined by the immunoreactive score (IRS) according to Remmele and Stegner (Preissner et al., 2012), from four independent experiments. Negative (IRS = 0–2), low (IRS = 3–4), intermediate (IRS = 5–6), and high (IRS = 7–12).

NET marker IRS		Treated H720 (n = 4)	Treated H727 (n = 4)
SSTR2	Negative	0 (0%)	0 (0%)
	Low	0 (0%)	0 (0%)
	Intermediate	0 (0%)	2 (50%)
	High	4 (100%)	2 (50%)
SSTR5	Negative	0 (0%)	2 (50%)
	Low	1 (25%)	1 (25%)
	Intermediate	1 (25%)	0 (0%)
	High	2 (50%)	1 (25%)

Contrary to the results of the qPCR in PNET cell line NT-3 and BP-NET cell line H727 in terms of SSTR5 transcript levels (shown in Figure 1), here SSTR5 protein was not downregulated after BYL719 and BYL719 plus everolimus treatment.

### Effects on proliferation by lanreotide and PI3K/mTOR inhibitors BYL719 and everolimus: BYL719 and everolimus overcome resistance of BP-NET cells to lanreotide

**FIGURE 4:**
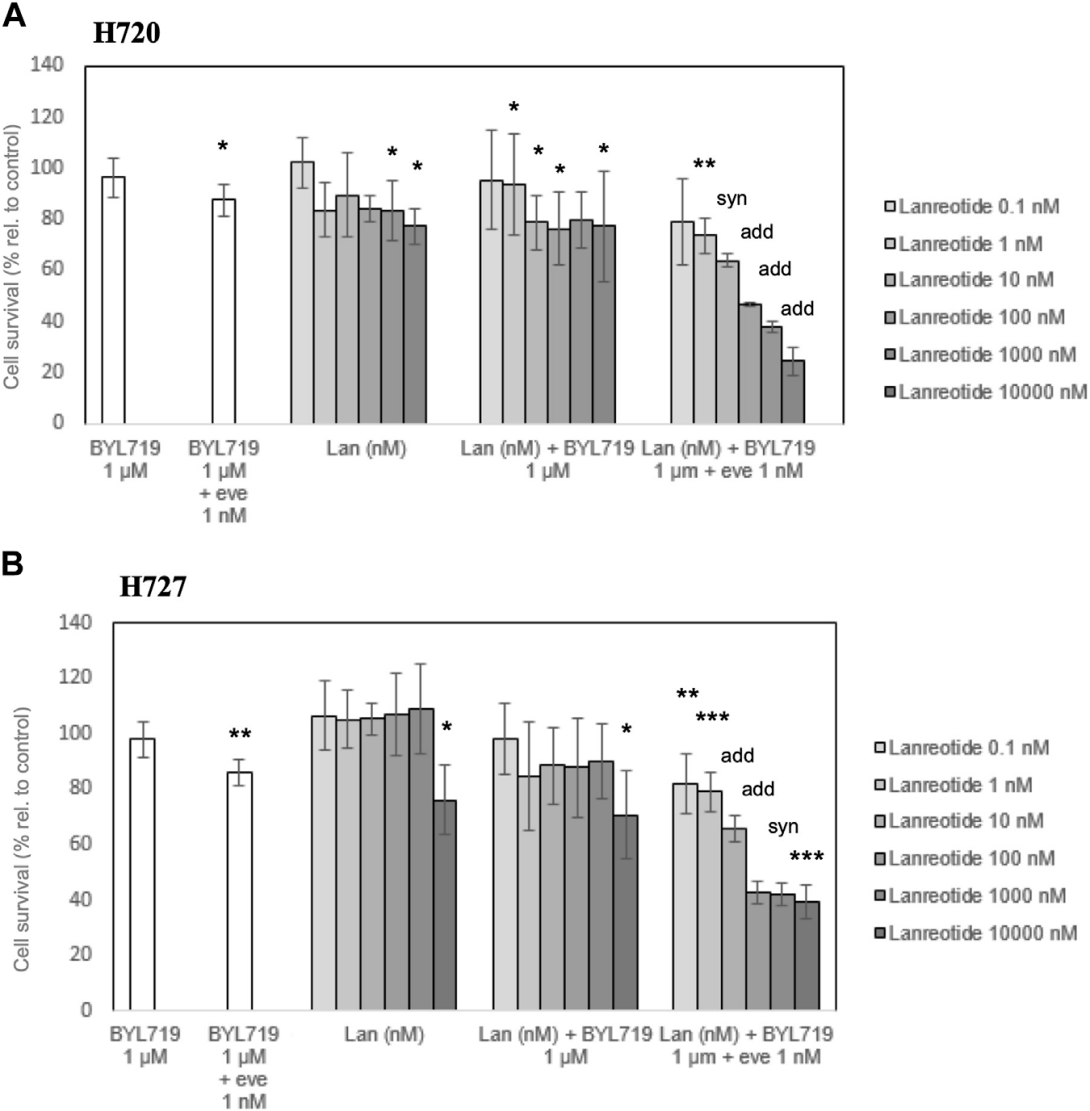
Survival analysis of H720 and H727 cells after 120-h treatment with (lan)reotide and 120-h treatment with lanreotide plus 48-h pretreatment. Pretreatment involves 48-h pre-incubation with 1 μM BYL719, or 1 μM BYL719 plus simultaneously 1 nM everolimus (eve). **(A,B)**: A WST-1 proliferation assay was done to measure cell survival. Data are presented as mean cell survival percentage ±SD of at least three independent experiments with five replicates per data point, relative to the untreated control. add: additive (combination effect compared to single lanreotide dose) (*p* ≤ 0.05); syn: synergistic (combination effect compared to single lanreotide dose) (*p* ≤ 0.01); *, **, ***: significant, very significant and extremely significant, compared to the control (*p* ≤ 0.05; *p* ≤ 0.01; *p* ≤ 0.001).

The potential antiproliferative effect of lanreotide at different concentrations was evaluated for 120 h in the H720 and H727 cell lines. WST-1 assays revealed a modest inhibition of proliferation when applying 1,000 nM and 10,000 nM lanreotide in H720 cells, and 10,000 nM in H727 cells. Targeting PI3K with selective inhibitor BYL719 at 10 μM for 48 h, prior to exposure to lanreotide, did not produce any additive or synergistic effect on proliferation inhibition. Targeting both PI3K and mTOR with a pretreatment consisting of 48-h pre-incubation with 1 μM BYL719 and 1 nM everolimus, prior to exposure to lanreotide, led to an important dose-dependent decrease in proliferation of both cell lines. In cell line H720 there was a synergistic antiproliferative effect in the combination BYL719 and everolimus at 10 nM lanreotide, and an additive effect at 100 nM, 1,000 nM and 10,000 nM. In cell line H727 there was an additive antiproliferative effect in the combination BYL719 and everolimus at 10 nM and 100 nM lanreotide, and a synergistic effect at 1,000 nM. This triple drug exposure reduced the amount of living cells by 20%–70%, whereas lanreotide alone or lanreotide plus BYL719 reduced it only by 1%–23% (shown in Figure 4).

**FIGURE 5:**
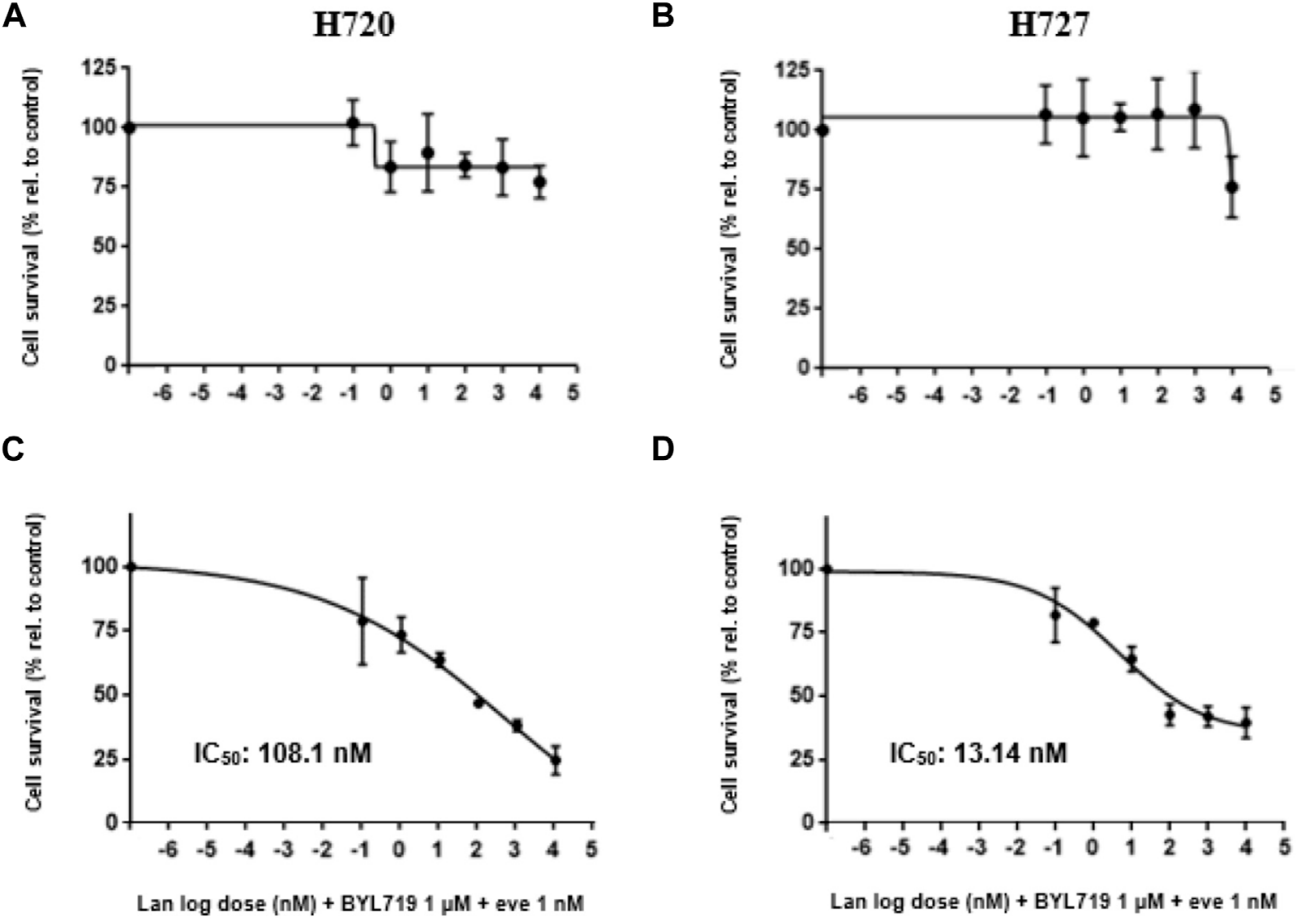
Dose-response curves for combination treatment of H720 and H727 cells against cell proliferation. **(A**–**D)**: Treated cells underwent a 48-h pretreatment with 1 μM BYL719 and 1 nM everolimus (eve) plus a 120-h treatment with lanreotide (nM) at different concentrations, or only 120-h treatment with lanreotide (nM) without pretreatment. Control cells received vehicle DMSO. A WST-1 proliferation assay was done to measure cell survival and the corresponding IC_50_ was calculated with GraphPad Prism software. IC_50_ could not be calculated for cells treated only with lanreotide due to inability of the SSA to reduce cell survival by 50% or beyond. Data are presented as mean relative cell survival ±SD of at least three independent experiments with five replicates per data point, *versus* the vehicle control.

The concentration of half-maximal effect (relative IC_50_) was determined for our best combination treatment: 1 μM BYL719 and 1 nM everolimus pretreatment prior to lanreotide exposure (at different concentrations) under the same experimental conditions based on WST-1 data (shown in Figures 5A,B). We obtained a higher IC_50_ value in H720 cells (108.1 nM) than in H727 (13.14 nM) (shown in Figures 5C,D). Accordingly, H727 cells showed a higher sensitivity to the combination treatment than H720 cells.

### Combination treatment of BYL719 and everolimus with lanreotide IC_50_ reduces cell division rate

**FIGURE 6:**
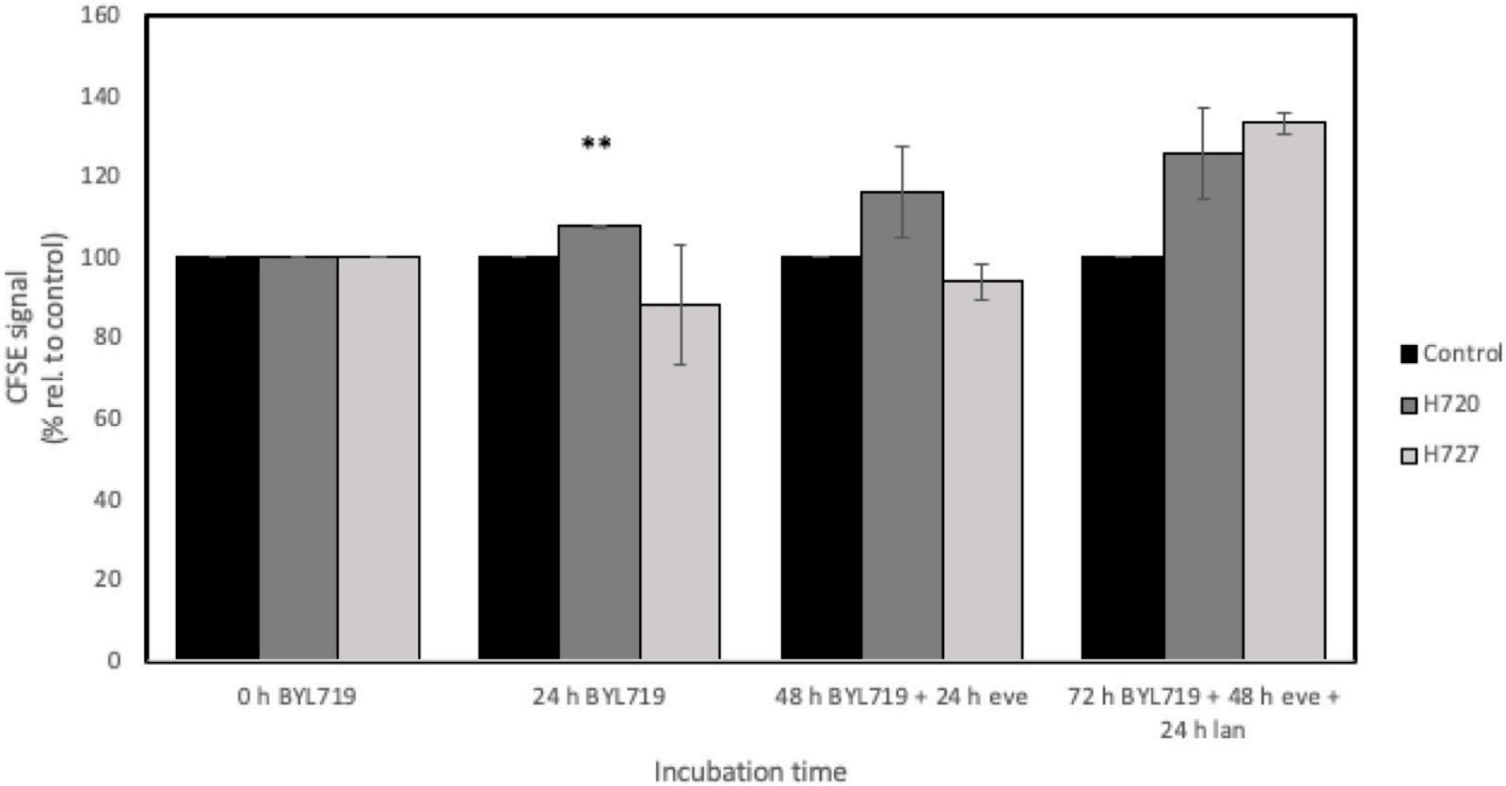
CFSE-based cell division assay of H720 and H727 cells with four timepoints for measurements during a 72-h long treatment. Treatment involved adding 1 μM BYL719 at timepoint 0 h, 24 h later 1 nM everolimus (eve) was added, and finally 24 h later IC_50_ lanreotide (lan) was added. Data are presented as mean CFSE signal ±SD of two independent experiments, with two replicates per data point, relative to the growth control. *, **, ***: significant, very significant and extremely significant, compared to the growth control (*p* ≤ 0.05; *p* ≤ 0.01; *p* ≤ 0.001).

H720 and H727 cells were labeled with CFSE and treated with a combination of inhibitors for 72 h in order to investigate their effect on cell division. IC_50_ doses of lanreotide specific to each cell line were used in this assay. CFSE signal was monitored at the beginning of the experiment and every 24 h until having measured four timepoints. At each timepoint of measurement, an (additional) inhibitor has been added for 24 h. The results from this analysis are shown in Figure 6.

The reduction in fluorescent signal due to cell division was markedly observed in the growth controls and set as the baseline, while the samples subjected to the different treatments generally displayed a stronger signal, implying that cell division had been hindered. In H727 cells, it is worth noting that only the addition of all three inhibitors (BYL719, everolimus and lanreotide) hindered cell division.

### Immunodetection reveals expression alteration of key proteins after combination treatment

Exemplary bands are shown in Figure 7. Please note that uncropped images associated with the Western blot have been included in the Supplementary Material (shown in Supplementary Figures S1–18). Due to the long list of proteins being assessed in this experiment, and because many of the samples were precious and needed for multiple antibodies, we have often cut the blots before performing any primary antibody incubations. This explains why there may be blots which are fragments rather than full-size blots.

**FIGURE 7:**
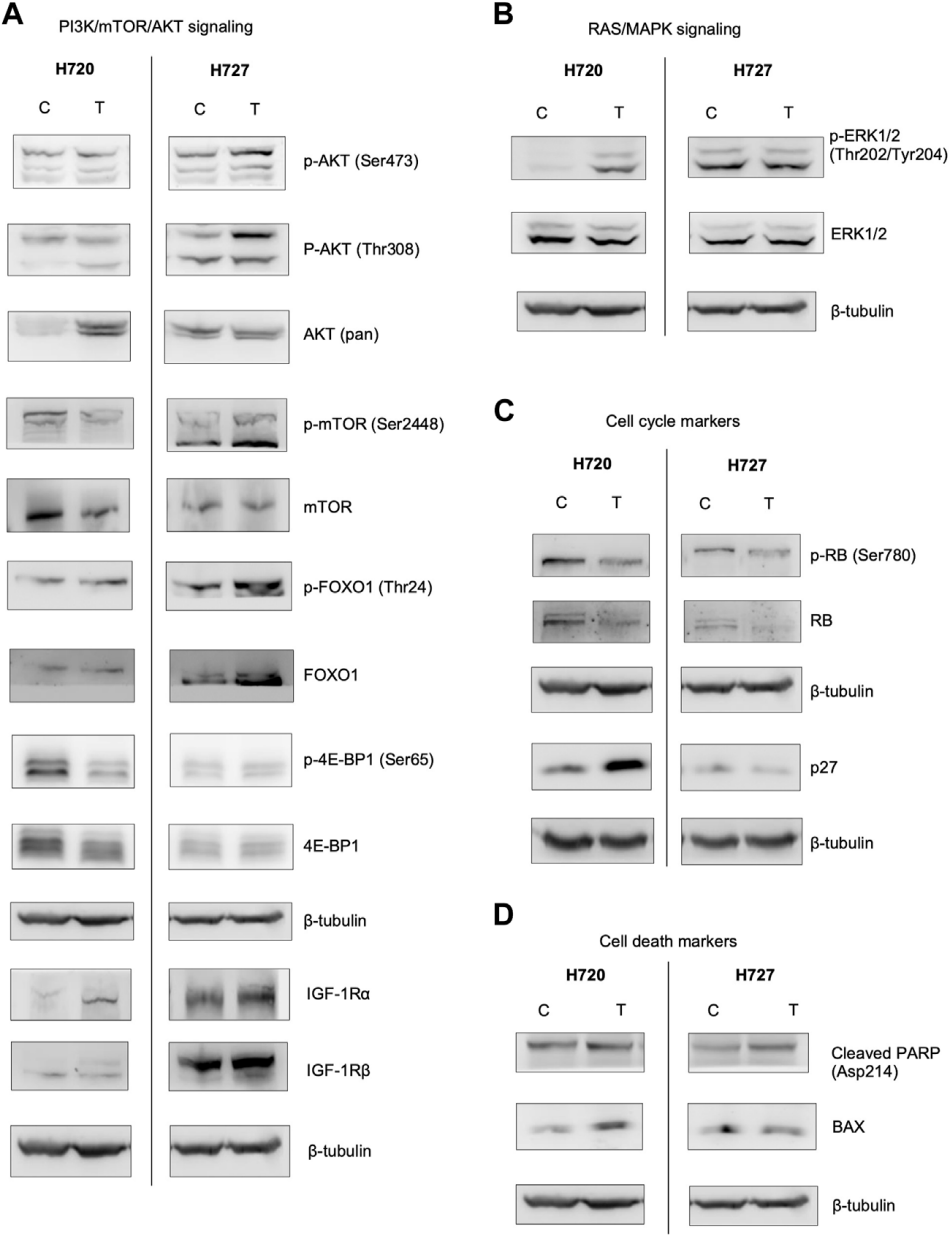
Protein immunodetection for the investigated signaling pathways, cell cycle and cell death markers in H720 and H727 cells. Cell lines were pre-treated with 1 μM BYL719 and 1 nM everolimus 48 h prior to 24-h exposure to lanreotide (IC_50_) (T), and compared with vehicle control (C). Images represent three independent Western blot-immunodetection experiments (except mTOR, AKT (pan) and cleaved PARP with two independent experiments for each). β-tubulin was used as loading control. Uncropped blots are available in the online Supplementary Material. **(A)**: Analysis of proteins and their phosphorylations downstream from PI3K. **(B)**: Analysis of ERK1/2, mediator of the RAS/MAPK pathway. **(C)** Analysis of the cell cycle markers. Treatment caused a decrease in Rb and phospho-Rb chemiluminescence. **(D)**: Analysis of cell death markers. Treatment caused an increase in cleaved PARP and BAX.

Detailed data from the band quantification analysis are listed in Table 3.

**TABLE 3:** Quantification of Western blot results analyzing signaling pathways and cell cycle and cell death in H720 and H727 cells after combination treatment. Cell lines were pretreated with 1 μM BYL719 and 1 nM everolimus 48 h prior 24-h exposure to lanreotide (IC_50_). Protein lysates were blotted for immunodetection and densitometric data was obtained. Band densities were normalized to that of the loading control. Data are shown as mean percentage of protein band density relative to the vehicle control ±SEM, from at least three independent experiments (except mTOR, AKT (pan) and cleaved PARP with two independent experiments for each). This is also expressed between brackets in the following way: –, – –, – – – means decreased band density vs. control; +, + +, + + + means increased band density vs. control; 0 means no difference from control; * means significant difference from control (*p* ≤ 0.05).

	H720	H727
	BYL719 + everolimus + lanreotide	
*PI3K/mTOR/AKT signaling*		
p-AKT (Ser473)	98.0% ± 29.1% (0)	99.8% ± ± 0.4% (0)
p-AKT (Thr308)	97.2% ± 41.4% (0)	97.9% ± 30.6% (0)
AKT (pan)	118.0% ± 21.1% (+*)	92.6% ± 3.9% (0)
p-mTOR (Ser2448)	94.4% ± 12.6% (0)	85.2% ± 29.5% (−)
mTOR	94.7% ± 26.2% (0)	55.6% ± 15.3% (-- *)
p-FOXO1 (Thr24)	107.9% ± 33.5% (0)	111.8% ± 33.0% (+)
FOXO1	118.0% ± 24.8% (+*)	118.8% ± 31.3% (+*)
p-4EB-P1 (Ser65)	57.9% ± 26.6% (-- *)	93.8% ± 35.3% (0)
4EB-P1	67.0% ± 28.7% (--)	89.5% ± 30.4% (−)
IGF-1Rα	112.4% ± 25.8% (+)	191.2% ± 32.2% (+++)
IGF-1Rβ	84.6% ± 25.5% (−)	97.9% ± 24.6% (0)
*RAS/MAPK signaling*		
p-ERK1/2 (Thr202/Tyr204)	85.7% ± 20.3% (−)	115.9% ± 24.3% (+)
ERK1/2	76.2% ± 29.4% (−)	111.5% ± 22.5% (+)
*Cell cycle markers*		
p-RB (Ser780)	91.2% ± 37.4% (0)	71.1% ± 30.3% (-- *)
RB	84.5% ± 35.3% (−)	70.5% ± 25.6% (-- *)
p27	127.6% ± 52.4% (++)	62.7% ± 8.9% (-- *)
*Cell death markers*		
Cleaved PARP (Asp 214)	116.3% ± 27.8% (+)	103.0% ± 30.4% (0)
BAX	131.8% ± 17.0% (++ *)	171.0% ± 37.2% (+++*)

PI3K/mTOR/AKT signaling. The aim of concomitant inhibition of PI3K/mTOR signaling via pretreatment with inhibitors BYL719 and everolimus is to avoid the limiting feedback activation of AKT. Therefore, we analyzed its direct targets mTORC1 and FOXO1, as well as their relevant phosphorylation sites (show in Figure 7A). In the slow-growing H727 we detected a significant decrease of mTOR and a significantincrease in FOXO1. In H720 cell line there was a significant increase in FOXO1 as well. In addition, we detected a significant increase of the AKT (pan) protein amount in H720 cells. 4E-BP1, a downstream target of mTOR, was also analyzed. H727 showed a slight decrease in 4E-BP1, while H720 showed both a significant, moderate decrease in p-4EB-P1 (Ser65) and a moderate decrease in 4E-BP1. The two subunits of IGF-1R, a modulator upstream of PI3K/AKT/mTOR, were analyzed. IGF-1Rα was highly increased in H720, while in H720 it was only slightly increased. IGF-1Rβ was slightly decreased in H720, while in H720 the densitometric data for the three experiments averaged null change vs. control (shown in Figure 7A).

RAS/MAPK Signaling. Here we found contrasting results. A slight decrease was observed in both p-ERK1/2 (Thr202/Tyr204) and ERK1/2 in H720 cells, whereas a slight increase was found for both protein forms in H727 (show in Figure 7B).

Cell cycle. A reduced band density of the negative cell cycle regulator Rb was detected in both cell lines. p-RB (Ser780) was significantly reduced in H727, however p27, a quiescence marker, was also significantly reduced. In H720, p27 was significantly increased (show in Figure 7C).

Cell death. Consistent with the cell survival assay (shown in Figures 7A,B), the analysis showed a significant increase in BAX in both cell lines after combination treatment. Cleaved PARP was also increased in H720 (show in Figure 7D).

### Multiplexed gene expression analysis of cancer-associated pathways in differentially treated H720 cells

Gene expression of relevant cancer-associated pathways (nCounter PanCancer Pathway Panel) were screened in the atypical lung carcinoid cell line H720 via NanoString technology (NanoString Technologies, United States) for three groups: 1) cells with 10 μM BYL719 48-h pretreatment prior lanreotide (IC_50_) 24-h treatment, 2) cells with 10 μM BYL719 48-h pretreatment only, and 3) vehicle controls.

**FIGURE 8:**
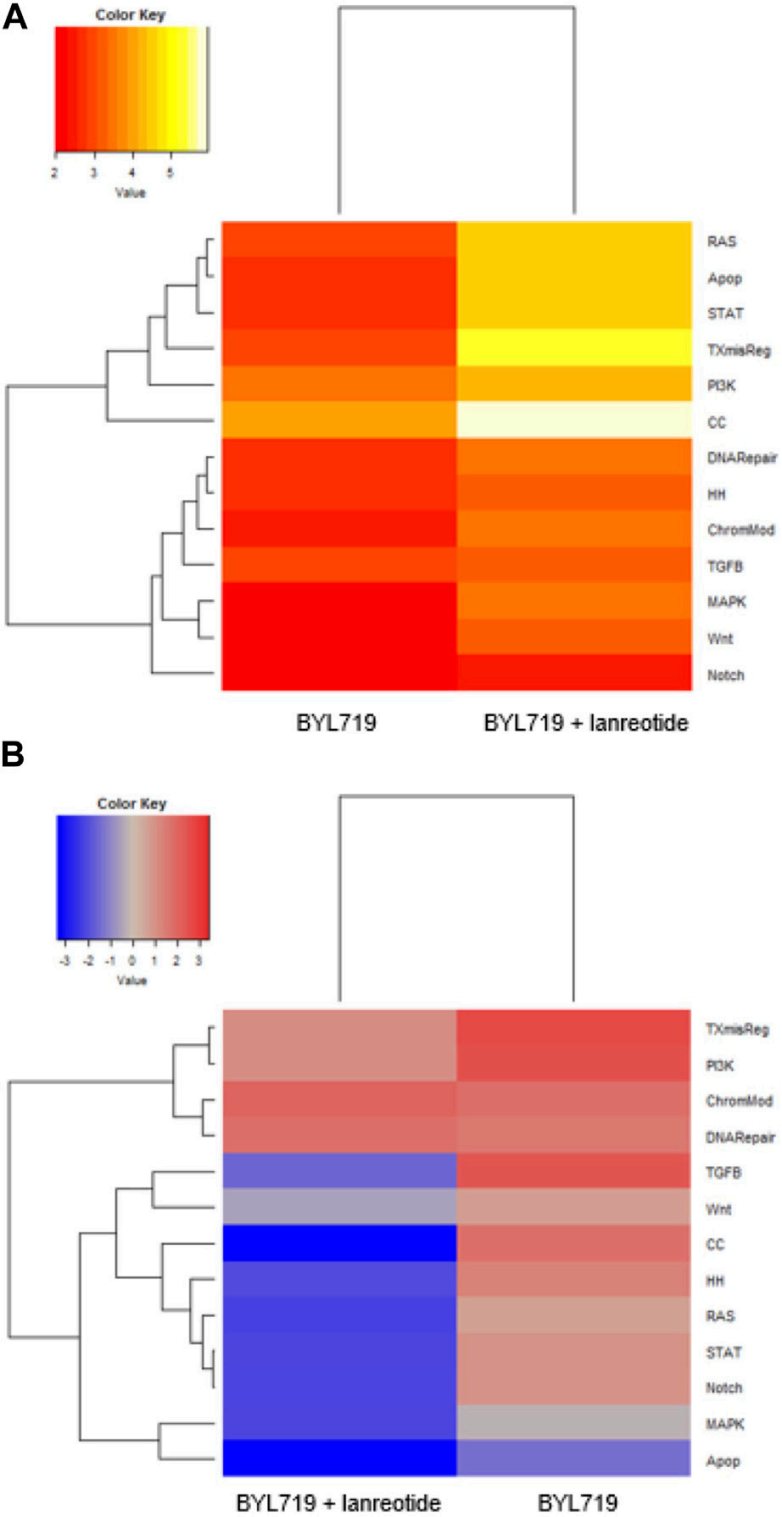
Gene expression analysis from cancer-associated pathways in treated H720. H720 cells received either 10 μM BYL719 pretreatment (48 h) plus lanreotide (IC_50_) treatment (24 h), or 10 μM BYL719 pretreatment (48 h) only. **(A)**: The heatmap displays the global significance scores of pathways after treatment and indicates that cell cycle regulation was the most affected mechanism. **(B)**: Directed global significance statistics measure the extent to which a pathway’s genes are up- or downregulated after treatment. Differentially upregulated pathways appear in red and differentially downregulated pathways appear in blue. This heatmap denotes that gene expression is predominantly affected in a negative way after combining BYL719 with lanreotide. Pathways: TXmisReg = transcriptional misregulation, PI3K = phosphoinositide 3-kinase, ChromMod = chromatin remodeling, DNARepair = DNA repair, TGFβ = transforming growth factor β, Wnt = wingless-related integration site, CC = cell cycle, HH = hedgehog, RAS = rat sarcoma, STAT = signal transducers and activators of transcription, Notch, MAPK = mitogen-activated protein kinase, Apop = apoptosis.

We selected all genes with fold change lower than 0.5 and greater than 1.5, combined with *p*-values lower than 0.05, to discover major differentially expressed genes. We clustered the relevant genes to pathways and calculated a pathway score (shown in Figure 8). We listed the genes affected by treatments and their log fold change intensity in Table 4. In summary, BYL719 plus lanreotide significantly reduced the expression level of genes which were already affected by monotreatment with BYL719. Furthermore, the BYL719 plus lanreotide combination affected a wider range of genes than BYL719 only. Notably, the strongest effect in pathway deregulation could be observed in the pathway for cell cycle regulation (show in Figure 8A), in which genes promoting cell cycle progression were downregulated (e.g., *CCNB1*, *CCNE1* and *CDKN2D*) (shown in Table 4). The rat sarcoma (RAS) pathway was also markedly deregulated and marker genes for cell proliferation were found to be downregulated (e.g., *FGF11*, *PKCA*, *MAP2K1*).

**TABLE 4:** Significantly altered transcripts after either BYL719, or BYL719 plus lanreotide treatment in H720 cells. (Panel A): Treatment-induced alterations in gene expression in H720 cells pretreated with 10 μM BYL719 (48 h) only. (Panel B): Treatment-induced alterations in gene expression in H720 cells pretreated with 10 μM BYL719 (48 h) prior to lanreotide (IC_50_) (24 h). Log fold change = < 0.5 or >1.5 after treatment, relative to covariate. FDR = false discovery rate. Pathways: TXmisReg = transcriptional misregulation, PI3K = phosphoinositide 3-kinase, ChromMod = chromatin remodeling, DNARepair = DNA repair, TGFβ = transforming growth factor β, Wnt = wingless-related integration site, CC = cell cycle, RAS = rat sarcoma, STAT = signal transducers and activators of transcription, Notch, MAPK = mitogen-activated protein kinase, Apop = apoptosis.

Gene ID Panel B	Log_2-_ fold change	*p*-value	FDR	Pathways
BAIAP3	0.61	3.76E-02	1.00E+00	TXmisReg
CCNE1	−0.63	1.93E-02	1.00E+00	PI3K, CC
CDC25A	−0.60	8.43E-04	3.93E-01	CC
CDKN1C	1.56	2.61E-05	4.43E-02	CC
CDKN2D	−0.61	7.30E-03	7.37E-01	CC
CHEK2	0.85	4.19E-02	1.00E+00	CC
CREB5	0.72	4.05E-03	6.46E-01	PI3K
EFNA5	0.62	1.50E-02	9.47E-01	PI3K, RAS
ERBB2	0.86	8.24E-03	7.55E-01	
FGF11	−0.82	4.75E-02	1.00E+00	MAPK, PI3K, RAS
IGF1R	1.58	2.00E-04	1.88E-01	TXmisReg, PI3K, RAS
ITGA3	0.66	3.13E-05	4.43E-02	PI3K
KIT	0.67	1.46E-02	9.47E-01	PI3K, RAS
LEFTY1	0.97	7.16E-03	7.37E-01	TGFβ
LIG4	−0.68	5.63E-03	6.63E-01	DNARepair
MAP3K1	0.71	6.16E-03	6.79E-01	MAPK
MED12	0.67	3.60E-02	1.00E+00	
MYCN	1.02	5.15E-03	6.61E-01	TXmisReg
TLX1	1.32	3.15E-03	6.46E-01	TXmisReg
Gene ID Panel B	Log_2-_ fold change	*p*-value	FDR	Pathways
AKT3	0.92	4.51E-03	2.83E-01	MAPK, STAT, PI3K, RAS, Apop
CACNG4	1.98	1.50E-02	4.29E-01	MAPK
CACNG6	1.07	6.29E-03	3.08E-01	MAPK
CASP8	−0.61	7.20E-03	3.23E-01	Apop
CCNB1	−0.75	3.35E-04	9.80E-02	CC
CCNE1	−0.76	5.93E-03	3.06E-01	PI3K, CC
CDC25A	−0.65	3.22E-04	9.80E-02	CC
CDKN1A	−1.56	6.31E-03	3.08E-01	TXmisReg, PI3K, CC
CDKN1C	1.36	2.35E-05	2.22E-02	CC
CDKN2D	−0.95	7.48E-04	1.30E-01	CC
CHEK2	0.73	3.99E-02	7.84E-01	CC
COL2A1	−0.66	3.27E-02	6.90E-01	PI3K
COL4A5	0.62	6.86E-03	3.23E-01	PI3K
DUSP6	1.46	9.55E-03	3.67E-01	TXmisReg, MAPK
FANCL	0.63	8.31E-04	1.30E-01	DNARepair
FGF11	−0.91	2.10E-02	5.39E-01	MAPK, PI3K, RAS
FOS	−0.73	4.42E-03	2.83E-01	MAPK
HOXA9	0.62	3.57E-02	7.37E-01	TXmisReg
HSPB1	−0.63	2.05E-02	5.31E-01	MAPK
IDH1	−0.71	1.77E-03	1.85E-01	
IGF1R	1.94	4.70E-05	3.32E-02	TXmisReg, PI3K, RAS
IL20RA	0.60	1.52E-03	1.65E-01	STAT
IRS1	0.65	4.69E-02	8.55E-01	PI3K
ITGA2	0.89	1.49E-02	4.29E-01	PI3K
JAG2	−0.78	3.35E-03	2.55E-01	Notch
LIG4	−0.92	9.77E-04	1.32E-01	DNARepair
MAP2K1	−0.64	7.51E-03	3.24E-01	MAPK, PI3K, RAS
MAPK8IP1	−0.59	3.17E-03	2.49E-01	MAPK
MAPT	−0.78	1.46E-02	4.29E-01	MAPK
MLF1	0.91	1.07E-02	3.78E-01	TXmisReg
MYCN	1.10	2.10E-03	2.12E-01	TXmisReg
PBX1	0.78	2.76E-04	9.80E-02	TXmisReg
PIK3R3	−0.77	1.11E-04	5.21E-02	STAT, PI3K, RAS, Apop
PPARGC1A	−0.86	2.55E-03	2.28E-01	ChromMod
PRKCA	−0.87	2.78E-02	6.37E-01	Wnt, MAPK, PI3K, RAS
RPS27A	0.74	4.02E-03	2.71E-01	DNARepair
RPS6KA6	−0.59	8.95E-04	1.32E-01	MAPK
STAT1	−0.79	6.07E-03	3.06E-01	STAT
THEM4	0.67	2.61E-02	6.14E-01	PI3K
TLX1	0.72	1.50E-02	4.29E-01	TXmisReg
TNFSF10	1.32	3.78E-02	7.74E-01	Apop

## Discussion

### Drug combination strategies to combat neuroendocrine tumors

Somatostatin analogues (SSAs) are already being used in clinical practice to treat hormone excess syndromes associated with neuroendocrine tumor disease. However, the antiproliferative role in bronchopulmonary neuroendocrine tumors (BP-NETs) is debated, given the limited available data (Tabaksblat et al., 2015). Due to existing evidence in gastroenteropancreatic NETs (GEP-NETs), SSAs are often used as first-line therapy in patients with advanced BP-NETs (Fazio et al., 2017). SST action requires activation of somatostatin receptors (SSTRs) to induce cell cycle arrest (quiescence) or apoptosis, predominantly through regulation of phosphotyrosine phosphatase and mitogen-activated protein kinase (MAPK) activities (Florio, 2008).

Given the limited evidence on the antiproliferative role of SSAs in BP-NETs thus far, combining SSA-induced MAPK inhibition with inhibition of other NET-relevant signaling pathways needs to be considered. As PI3K signaling is often activated in NETs and has been implicated to play a critical role in tumorigenesis and aggressiveness of BP-NETs (Capodanno et al., 2012; Briest and Grabowski, 2014), a 2017 study assessed the effects of selective PI3K inhibition by the novel agent BYL719 (alpelisib) in PNET cells and BP-NET cells (Nölting et al., 2017). The authors reported an enhanced neuroendocrine differentiation in cells after treatment with BYL719, evident from the induction of chromogranin A and SSTR1/2 mRNA synthesis. They additionally tested the combination BYL719 plus everolimus and found that this combination was synergistic (through simultaneous PI3K/mTOR inhibition) in PNET cell lines BON-1 and QGP-1. The combination also significantly increased SSTR2 transcription even further. Here we assessed the effects of BYL719 and combination BYL719 plus everolimus as pretreatment to enhance the antiproliferative effects of the SSA lanreotide.

### PI3K inhibitor BYL719 greatly induced expression of SSTR2 in H727, but downregulated expression of SSTR5 in both H727 and NT-3 cells

PNET cell line NT-3 and BP-NET cell line H727 were incubated with selective PI3K inhibitor BYL719. This was done with the aim to reproduce the effects on SSTR mRNA transcription seen previously in PNET cell lines BON-1 and QGP-1 upon treatment with BYL719 (Nölting et al., 2017). While the increase in SSTR2 mRNA transcript levels was only minimal in NT-3 cells, H727 cells experienced a greater induction. Interestingly, SSTR5 seems to be differentially regulated by BYL719 compared to SSTR2, as evidenced by the results of the qPCR. This phenomenon was also observed previously (Nölting et al., 2017).

Fluorescence microscopy detecting induction of SSTR2 and SSTR5 protein production in H720 and H727 revealed that production of either was greater upon treatment with BYL719 only or BYL719 plus everolimus. This contradicts the reduction in SSTR5 transcripts levels previously determined by the qPCR. This apparent controversy could arise from the fact that here we determined protein production and mRNA levels at the same timepoint. A reduction in SSTR5 transcript could occur as a result of a compensatory mechanism: first, there is SSTR5 induction (evidenced by SSTR5 protein accumulation), and subsequently, there is a secondary transcriptional counterregulation (indicated by mRNA decrease). The effect of this secondary transcriptional counterregulation on protein production might have not been possible to detect at the timepoint in which cells were fixed for immunostaining, but possibly a few hours later. In addition, post-transcriptional management of mRNA, where the specific transcript gets degraded while its protein half-life increases, could also account for this result. In this scenario, the pool of already synthesized SSTR5 mRNA could be more efficiently translated and/or protein stability could increase, therefore increasing SSTR5 protein accumulation. It is worth to note, however, that reportedly the binding of lanreotide to SSTR2 is most predominant and crucial for its therapeutic effects to take place (Murray and Melmed, 2008).

All in all, taking into consideration that SSTR2 expression has been observed as a predictor of improved survival in NET patients, the SSTR2 reinduction effect described here could be of great therapeutical relevance. If this effect can be reproduced in BP-NET patients, this could mean that BYL719 as a mono agent and combination of BYL719 plus everolimus could be used to improve therapeutic and diagnostic applications that could benefit from SSTR reinduction, e.g., peptide receptor radionuclide therapy (PRRT), where SSAs are labeled with therapeutic radionuclides, and SSTR2-based imaging (Xu and Zhang, 2015). To expand on the relevance for imaging, SSTR2 reinduction could be particularly helpful in BP-NETs where SSTR2 expression is considerably low and would otherwise not make them a good candidate for this technique.

### Concomitant inhibition of PI3K/mTOR pathways enhances antiproliferative effects of lanreotide

Our results show that lanreotide decreases the cell number of human BP-NET cells H720 and H727 in a dose-dependent manner (0.1–10,000 nM) after pretreatment with BYL719 plus everolimus. This data is in line with a growing body of evidence showing that SSAs in combination with PI3K/mTOR inhibitors are capable of strongly inhibiting the mitogenic effect of growth factors in NETs, including bronchopulmonary NETs (Villaume et al., 2010; Fazio et al., 2013; Dogan Turacli et al., 2015; Pivonello et al., 2017). In preliminary experiments we incubated H720 and H727 with lanreotide only and found out that they were rather resistant to the action of the SSA (Jost-Brinkmann et al., 2016). This was also observed in NT-3 cells (unpublished data). Interestingly, a similar observation was found when researchers tested the SSA octreotide in 5 NET lines including H727, as the conclusion was that octreotide did not inhibit proliferation (Exner et al., 2018).

A previous study on H727 cells obtained a decrease in cell survival of more than 50% only at a starting dose of 100 nM (Fotouhi et al., 2016). Here we obtained lanreotide IC_50_ values of 13.4 nM for H727 and 108.1 nM for H720, in the scope of the combination with BYL719 and everolimus. We have observed that SSTR2 is upregulated, most probably due to this pretreatment, and it would be therefore understood that more receptors become available to mediate the antiproliferative effects of lanreotide, *in vitro*. To note, a 2013 study on H720 and H727 cells treated with dual PI3K/mTOR inhibitor BEZ235 revealed a greater reduction in cell viability in H720 cells compared to the H727 cells. The authors also mention finding higher expression levels in terms of mRNA in H720 cells vs. H727 cells (Gagliano et al., 2013). In our study we observed as well higher SSTR induction in H720 cells and a reduction in cell viability in both cell lines, however the greater reduction in cell viability was observed in H727. While it is true that the cell viability methods used in each study were different and the treatment in the present study involved not only PI3K/mTOR inhibitors but also lanreotide, it is also true that H720 cells are of atypical carcinoid origin. Thus, we may consider the existence of rare resistance mechanisms inducing a faster cell division, as a response to cell death pathways activation after lanreotide exposure.

The study of SSTR subtype induction through qPCR and immunocytochemistry after pretreatment with BYL719 and everolimus, together with the decrease in cell viability after cells were pretreated prior to lanreotide exposure, provided evidence that implicate SSTR2 and SSTR5 subtypes in the lanreotide-mediated antiproliferative effects. We suggest to further analyze this strategy of up-regulating SSTR protein expression and thereby sensitizing cells to the therapeutic actions of lanreotide *in vitro* and *in vivo*.

We acknowledge that the benefits shown here by lanreotide come most probably as a result of the concomitant inhibition of PI3K/mTOR pathways. This combined inhibition has been explored in the past as a strategy to circumvent the mechanisms of resistance observed in mTOR-resistant NETs. While the preclinical results have been favorable enough to bring the studies into humans, clinical studies using dual PI3K/mTOR inhibitor BEZ235 have been early terminated due to toxicity issues and modest efficacy (Fazio, 2015). The difficulty in translating the combination treatment proposed here, which includes the PI3K/mTOR concomitant inhibition and additionally lanreotide, into human application is clear. We consider, however, that there is growing evidence suggesting that tolerability can be improved when lowering doses or tweaking the frequency of drug administration to intermittent schedules, without comprising on the benefits (Ter Heine et al., 2018; Curigliano et al., 2021). The recent phase 1B study NTC02077933 particularly explored the safety and efficacy of BYL719 and everolimus with/without an aromatase inhibitor in patients with PNETs, reporting a manageable safety profile and encouraging efficacy in either treatment arm.

### Cancer-associated signaling pathways show alteration at the protein and transcript level after cell treatments

As mentioned previously for H720 cells, both p-ERK and ERK were decreased after combination treatment (BYL719 and everolimus plus lanreotide). The addition of lanreotide could possibly have resulted in the suppression of the MAPK feedback activation which, reportedly, monotreatment with BYL719 induces (Wong et al., 2015). Cell death appeared to be induced in both H720 and H727 cells lines, as suggested by the significant rapid increase in BAX after 48 h. Expression of tumor suppressor FOXO1 which is located downstream of PI3K/mTOR was induced after combination treatment in both cell lines as well. The degree of cell growth inhibition observed with this treatment corresponded with the level of suppression of p-RB in H727. Both mTOR and p-mTOR were decreased in H727 cells, with varying levels of significance. In summary, the Western blot analysis demonstrated a better anti-tumor effect in typical carcinoid H727 cells than in the more aggressive, atypical carcinoid H720 cells.

The effect of combined treatment in signaling pathways was more profoundly investigated in H720 cells. This was done by way of a multiplex gene expression analysis using the nCounter PanCancer Pathway Panel (NanoString Technologies, United States) which compared the three following groups: cells treated with BYL719 only, cells treated with BYL719 prior to lanreotide exposure, and cells receiving vehicle. The cell cycle signaling pathway was identified as having the greatest degree of differential downregulation after the combination treatment. Interestingly, apoptosis appeared to be less induced in cells subjected to combination treatment than cells treated only with BYL719. This could indicate that sustained cell cycle arrest plays a larger role in the reduction of tumor cell growth/viability than apoptosis. Accordingly, the Western blot for H720 subjected to the combination treatment showed an increase in p27, a marker for quiescence.

Notably, the NanoString analysis revealed an increase in IGF-1R mRNA levels (another gene from the RAS pathway). This result is in alignment with NETs in previous studies, attributed to a growth factor-mediated feedback loop after mTORC1 inhibition, which exerts compensatory mechanisms (Svejda et al., 2011; Nölting et al., 2017). IGF-1R activation seems to have an essential role in tumor cell proliferation of different tumor types (De Santi et al., 2016; Svalina et al., 2016; von Wichert et al., 2000), which has provided reason to incorporate anti-IGF-1R drugs in the frame of the disease management. Interestingly, a study running in parallel to this one found that an IGF-1R inhibitor significantly suppressed growth in H727 (Werner et al., 2019). Phase 3 clinical trials targeting the IGF axis have not been successful thus far, however there is consensus that combining anti-IGF-1R drugs with other biological therapies might be of major translational relevance, with potential for personalized medicine (Motylewska et al., 2020).

Additional potential targets have been identified reported in the literature in the past years such as COX-2 and CUX1 which mediate tumor progression via cell proliferation and angiogenesis (Gao et al., 2018; Krug et al., 2020). In light of this, we acknowledge the need to perform further studies to assess the effects of more combination therapies.

This study has potential limitations. The authors acknowledge the possibility of using reagent BrdU to examine cell proliferation as an alternative to the labeling methods used in the study, or to complement the WST-1 survival assay which was here performed. Combined application of both reagents has been done in other cell types (McCann and Imani, 2007; Yin et al., 2014). Additionally, in contrast to the Western Blot experiment, the Nanostring experiment did not include samples treated with the triple combination (BYL719 and everolimus plus lanreotide) and it was only performed on H720 cells. This was due to budgetary constraints regarding the use of Nanostring technology, the limited number of Nanostring sample slots available and shifting endpoints during the course of the study. In view of this, the authors consider that the results from this experiment in particular are difficult to extrapolate to other types of BP-NETs.

## Conclusion

The implications of the results of this study in cancer therapeutics could be quite meaningful, especially given that therapy options to treat NETs are limited. BP-NET cells undergoing pretreatment with BYL719 (alpelisib) and everolimus were found to become sensitized to the antiproliferative effects of lanreotide, possibly due to induction of SSTR expression. Notably, the induction of SSTR expression observed here could be exploited not only for this effect, but also to enhance other therapies, such as PRRT, and improve the imaging of BP-NETs (particularly the imaging of those which exhibit low/null SSTR expression). This could ultimately make way for a more personalized approach in disease management.

Additional studies will be of necessity to further verify the pharmacological actions of the three agents in combination, utilizing inhibitors in short and long-term experiments. Combinatory therapies like this or with a similar mechanism of action may be worth consideration in the treatment of other NETs.

## Data Availability

The original contributions presented in the study are included in the article/Supplementary Material, further inquiries can be directed to the corresponding author.
