# Supplementary material for: Concomitant inhibition of PI3K/mTOR signaling pathways boosts antiproliferative effects of lanreotide in bronchopulmonary neuroendocrine tumor cells
Source: Front Pharmacol. 2024 Feb 5;15:1308686. doi: 10.3389/fphar.2024.1308686 (PMC10875132; doi:10.3389/fphar.2024.1308686)
Supplement: Supplementary file 1 [file DataSheet1.PDF]

Supplementary Material

Concomitant Inhibition of PI3K/mTOR Signaling Pathways Boosts Antiproliferative Effects of Lanreotide in Bronchopulmonary Neuroendocrine Tumor Cells

Claus von Hessert-Vaudoncourt*, Sara Lelek, Christina Geisler, Teresa Hartung, Vanessa Bröker, Franziska Briest, Liliana Mochmann, Fabian Jost-Brinkmann, Dagmar Sedding, Joana Benecke, Helma Freitag, Sebastian Wolfshöfer, Hedwig Lammert, Svenja Nölting, Michael Hummel, Jörg Schrader and Patricia Grabowski

*** Correspondence:**

Corresponding author: Claus von Hessert-Vaudoncourt
E-mail: claus.vhessert@gmail.com

Supplementary Figures

Uncropped Western blots

Uncropped images associated with our Western blot data for this manuscript have been included in this section. Please note that due to the long list of proteins being assessed in this experiment, and because many of our samples were precious and needed for multiple antibodies, we have often cut the blot before performing any primary antibody incubations. This explains why there may be blots which are fragments rather than full-size blots.

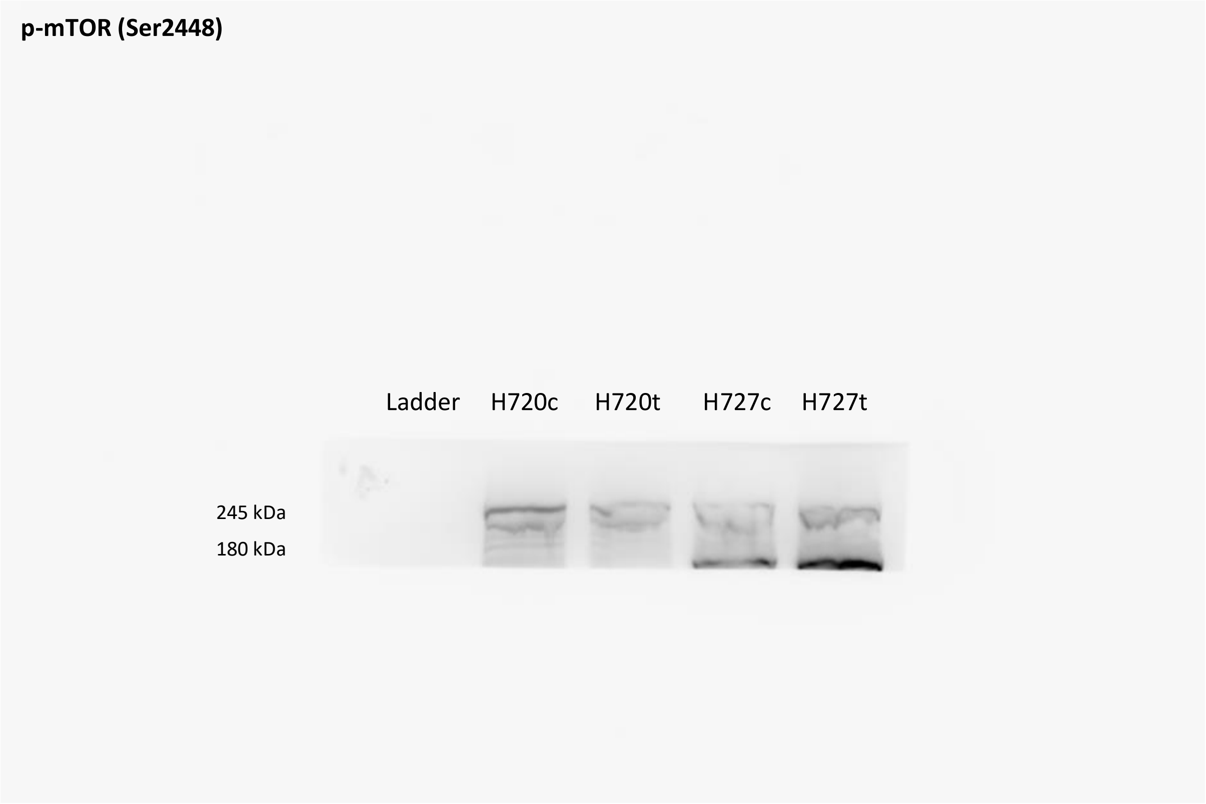

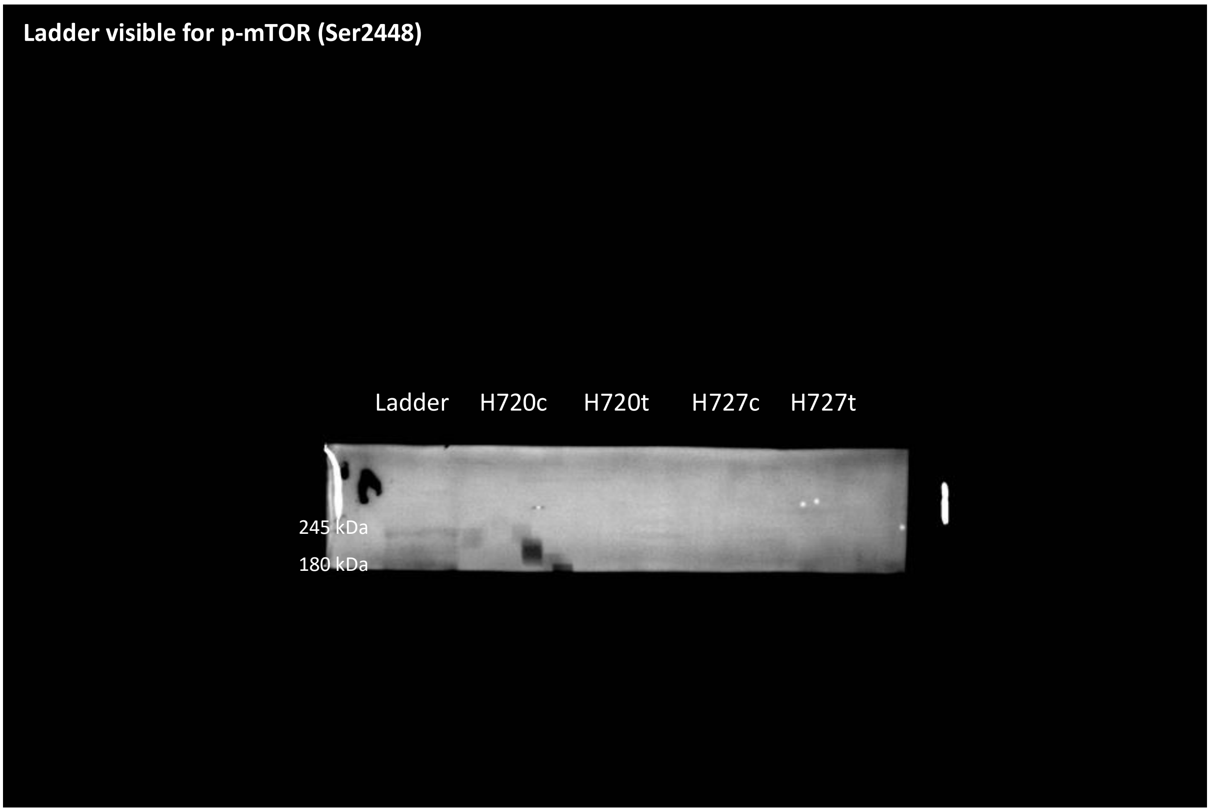

Supplementary Fig. 1. Uncropped image associated with Figure 7A: detection of p-mTOR (Ser2448). Ladder shown separately.
c = control
t = treated

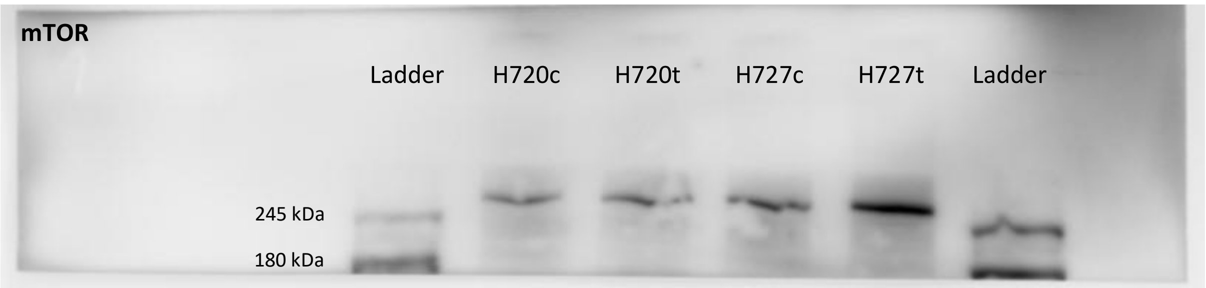

Supplementary Fig. 2. Uncropped image associated with Figure 7A: detection of mTOR.
c = control
t = treated

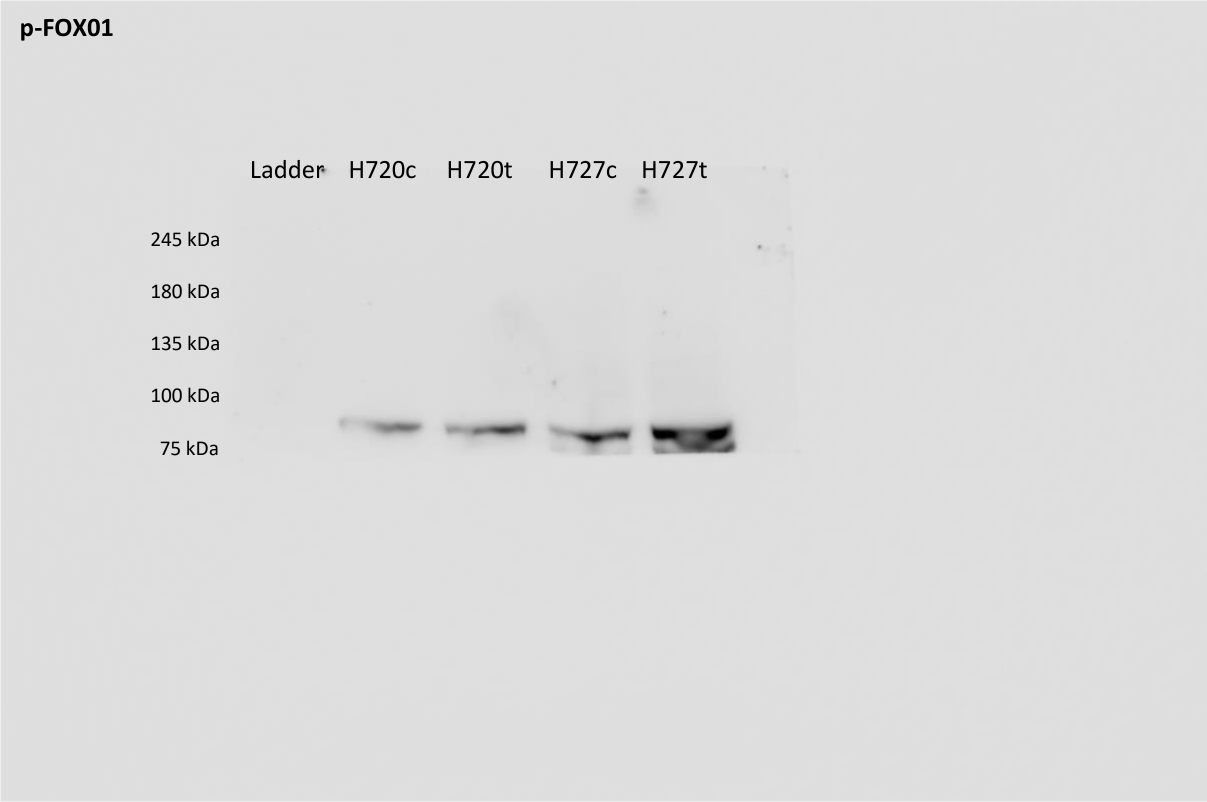

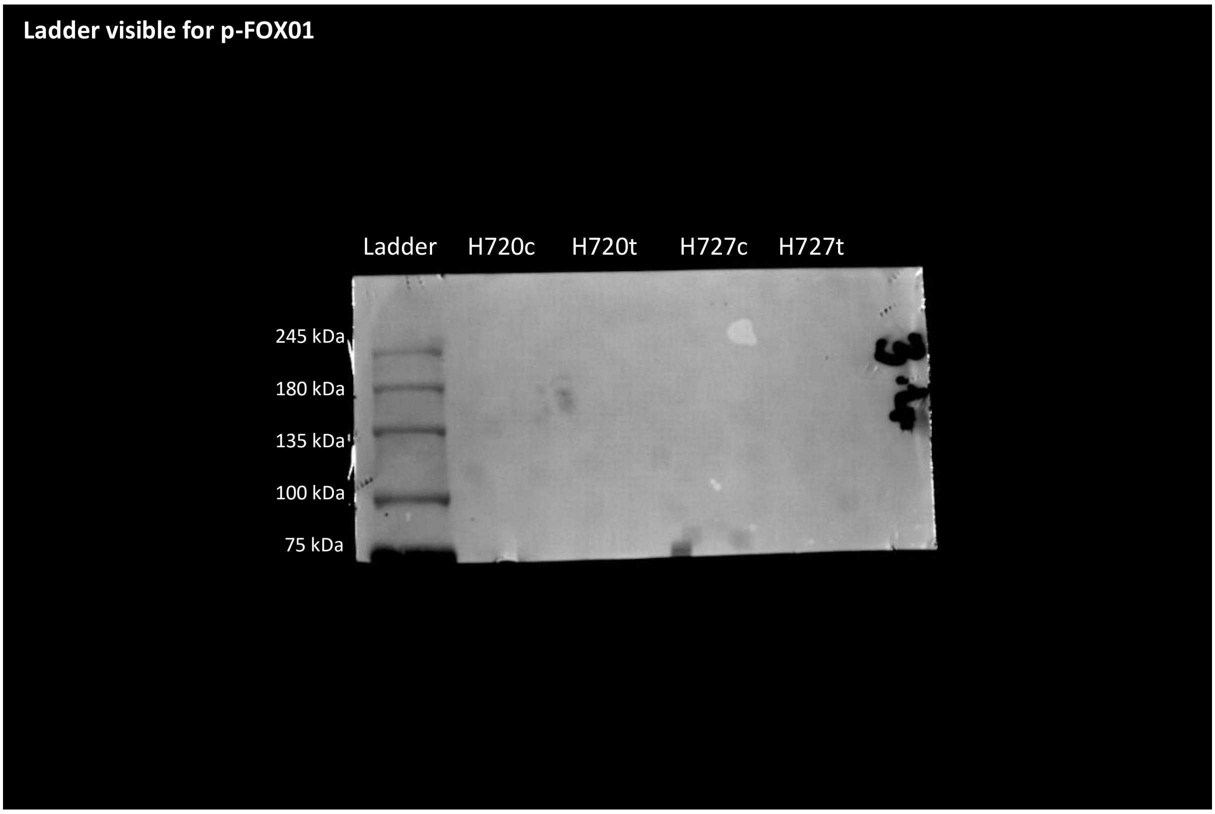

Supplementary Fig. 3. Uncropped image associated with Figure 7A: detection of p-FOXO1. Ladder shown separately.
c = control
t = treated

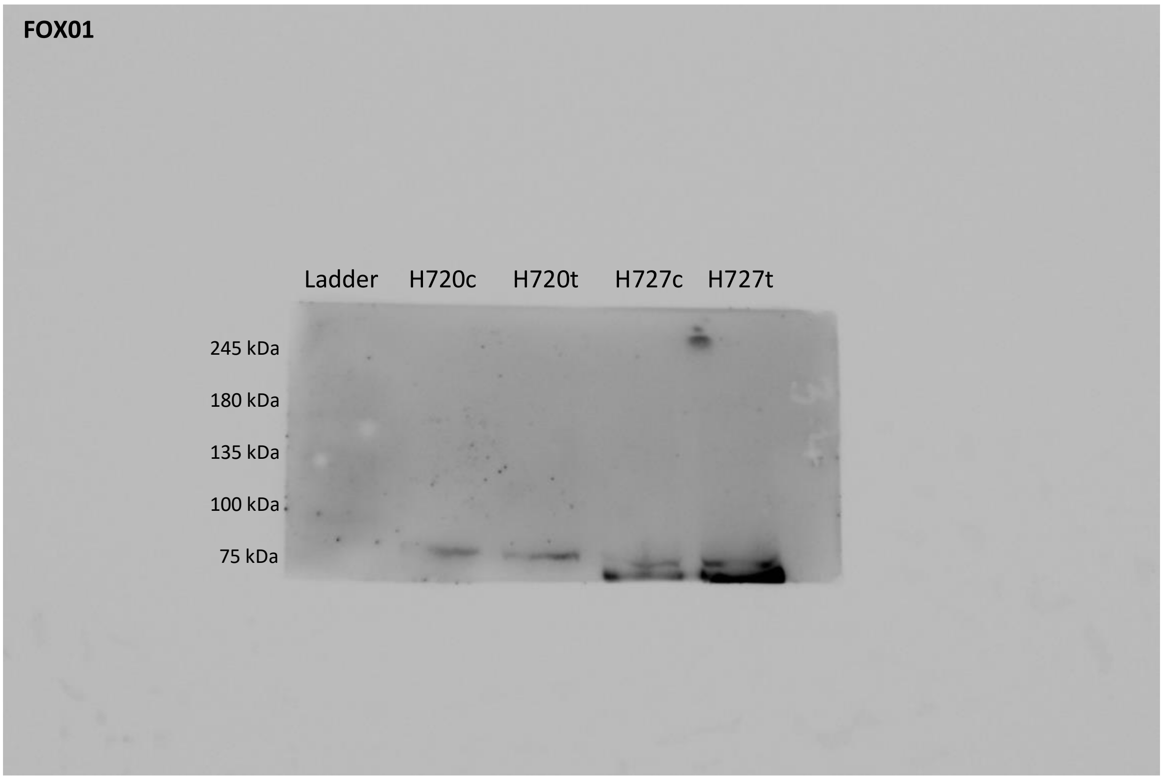

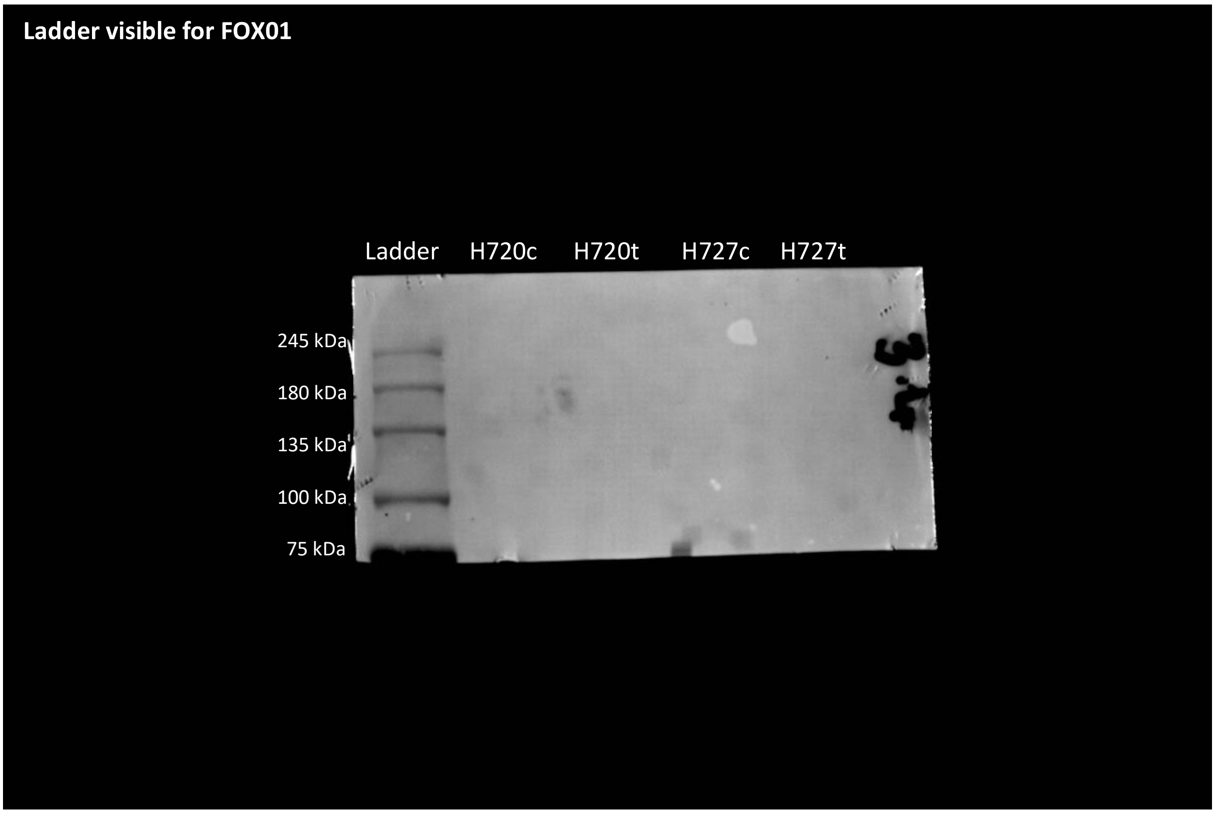

Supplementary Fig. 4. Uncropped image associated with Figure 7A: detection of FOX01. Ladder shown separately.
c = control
t = treated

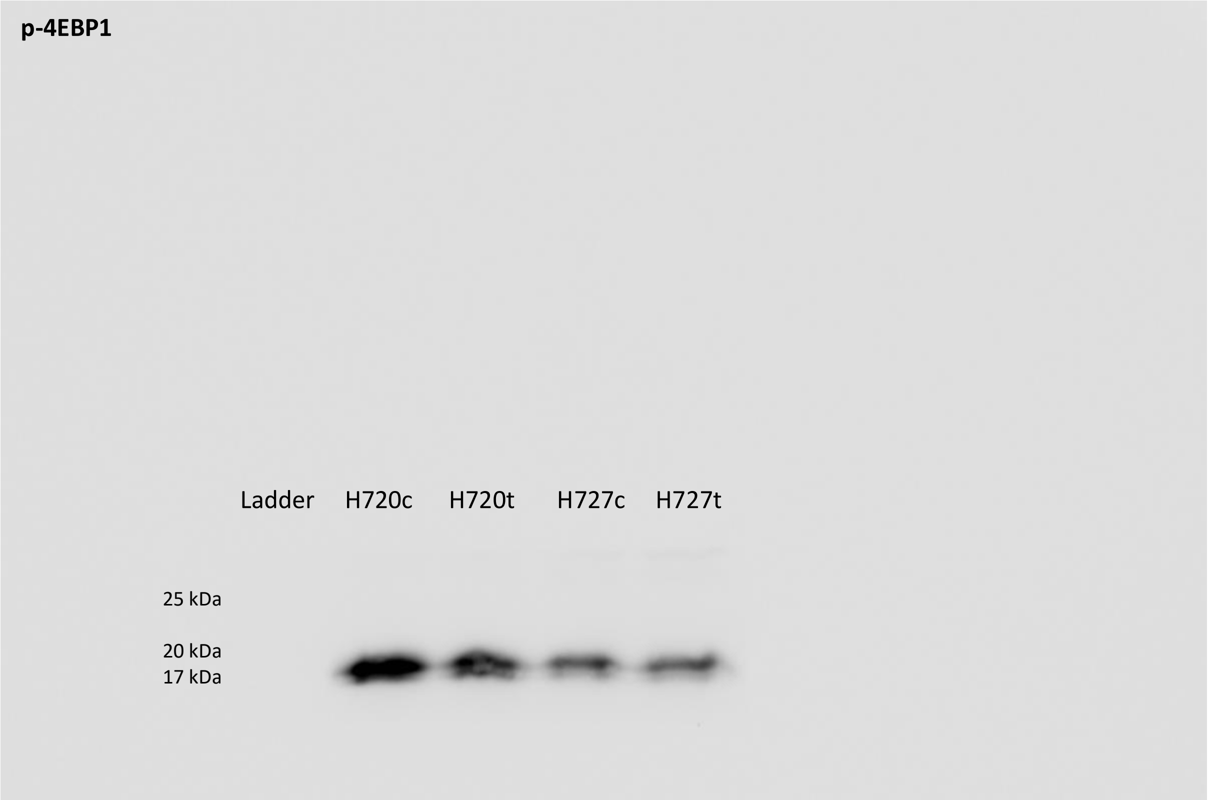

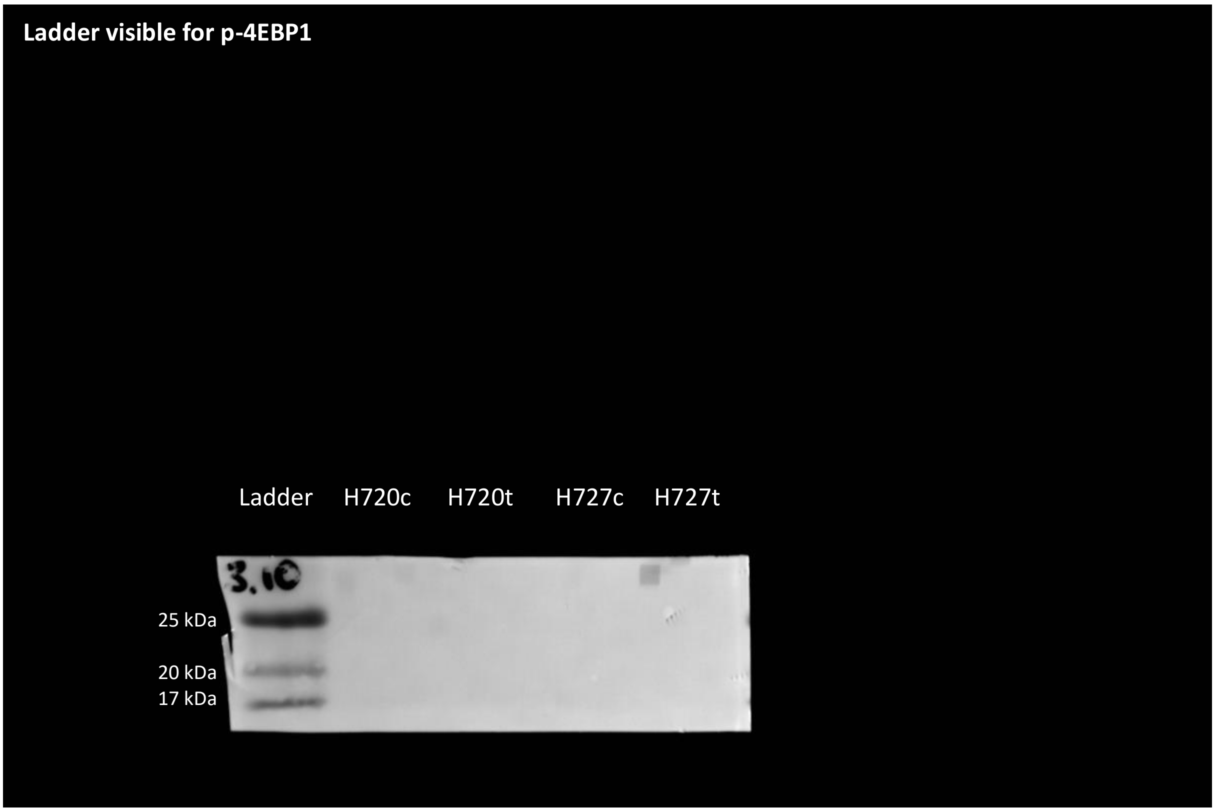

Supplementary Fig. 5. Uncropped image associated with Figure 7A: detection of p-4EBP1. Ladder shown separately.
c = control
t = treated

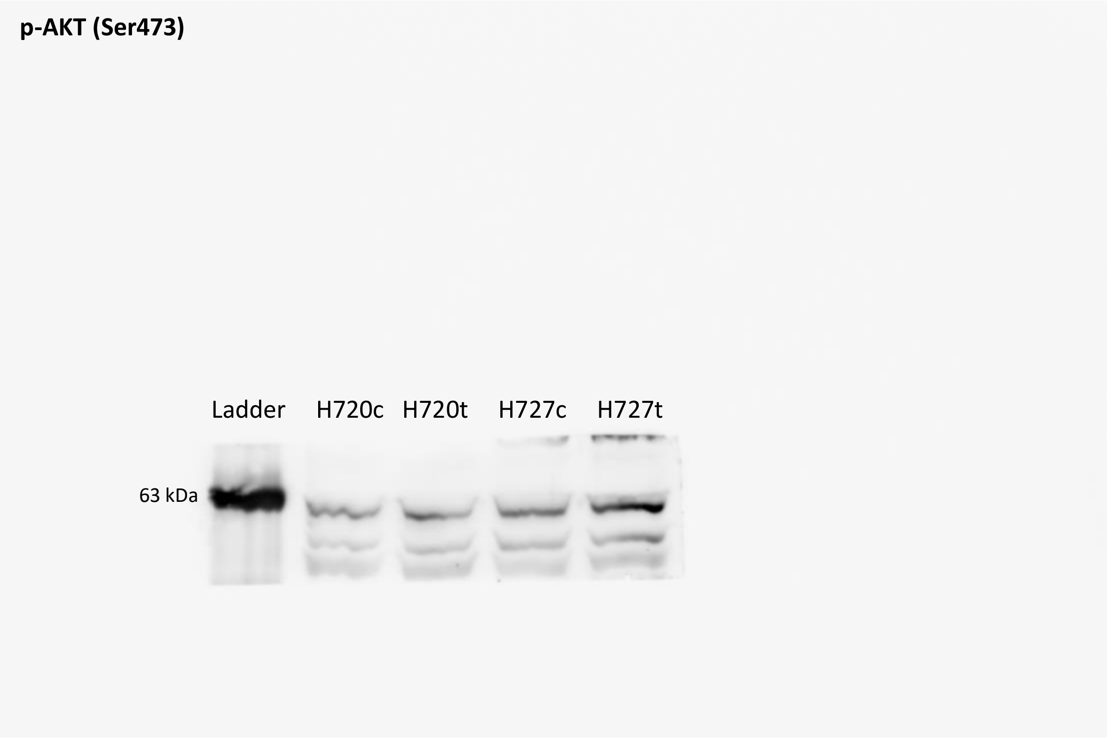

Supplementary Fig. 6. Uncropped image associated with Figure 7A: detection of p-AKT (Ser473).
c = control
t = treated

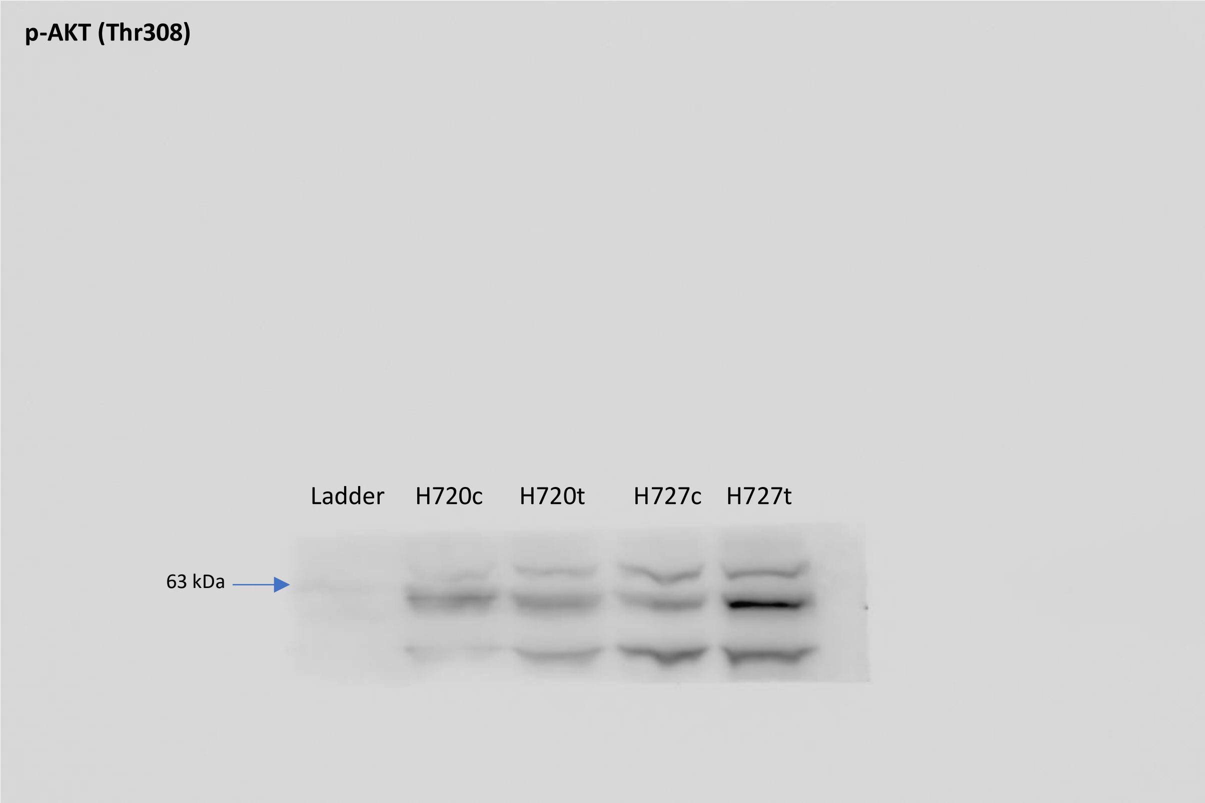

Supplementary Fig. 7. Uncropped image associated with Figure 7A: detection of p-AKT (Thr308).
c = control
t = treated

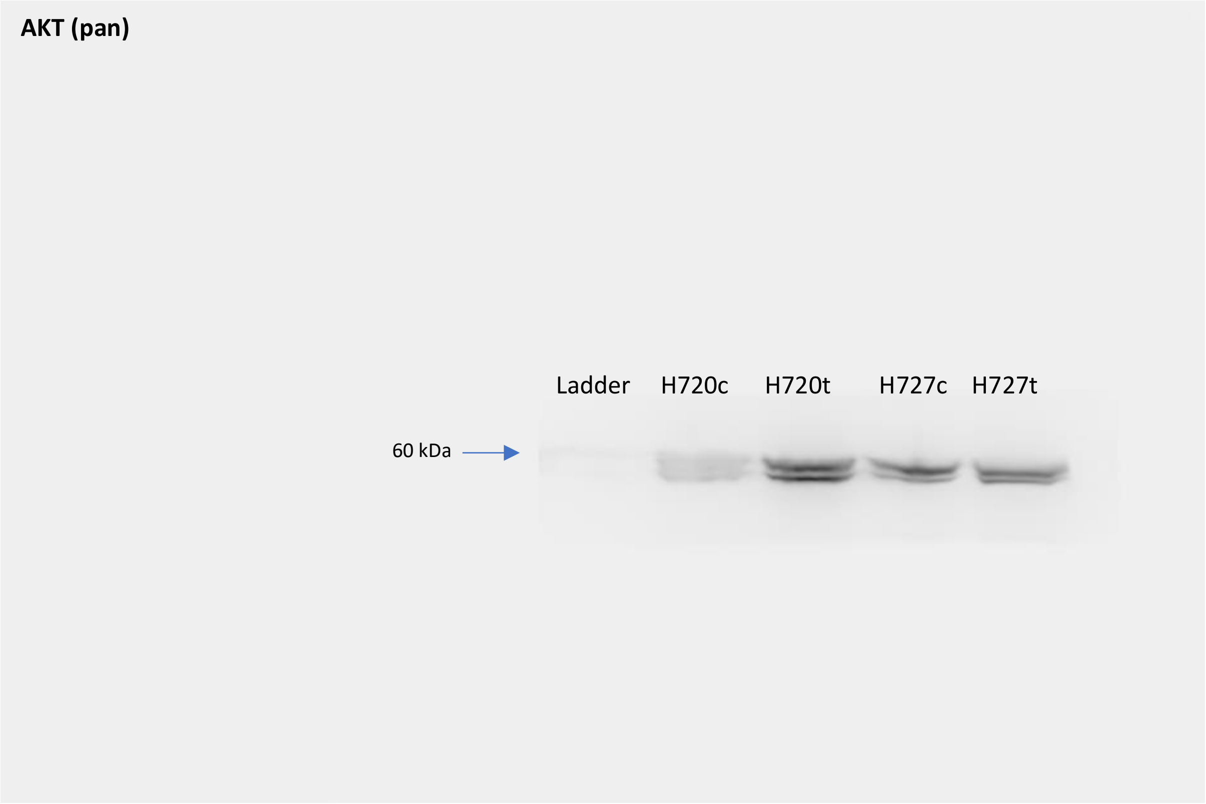

Supplementary Fig. 8. Uncropped image associated with Figure 7A: detection of AKT (pan).
c = control
t = treated

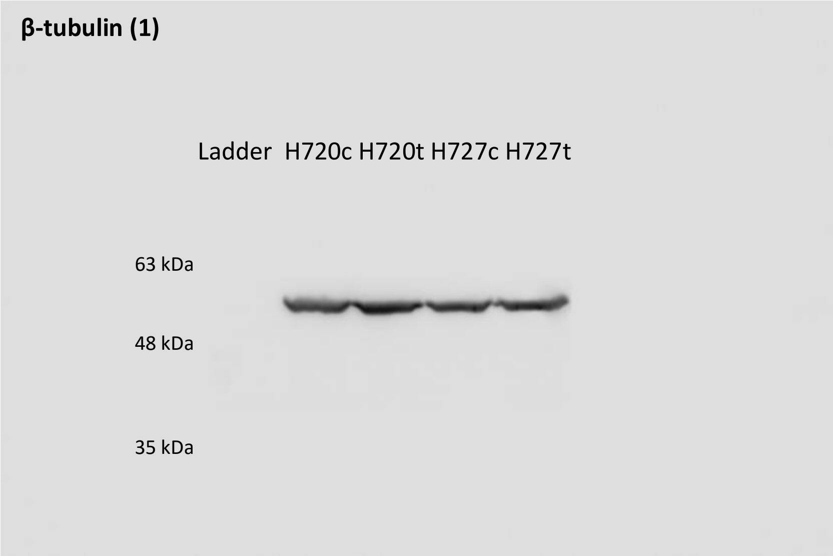
β-tubulin (1)
Ladder H720c H720t H727c H727t
63 kDa
48 kDa
35 kDa

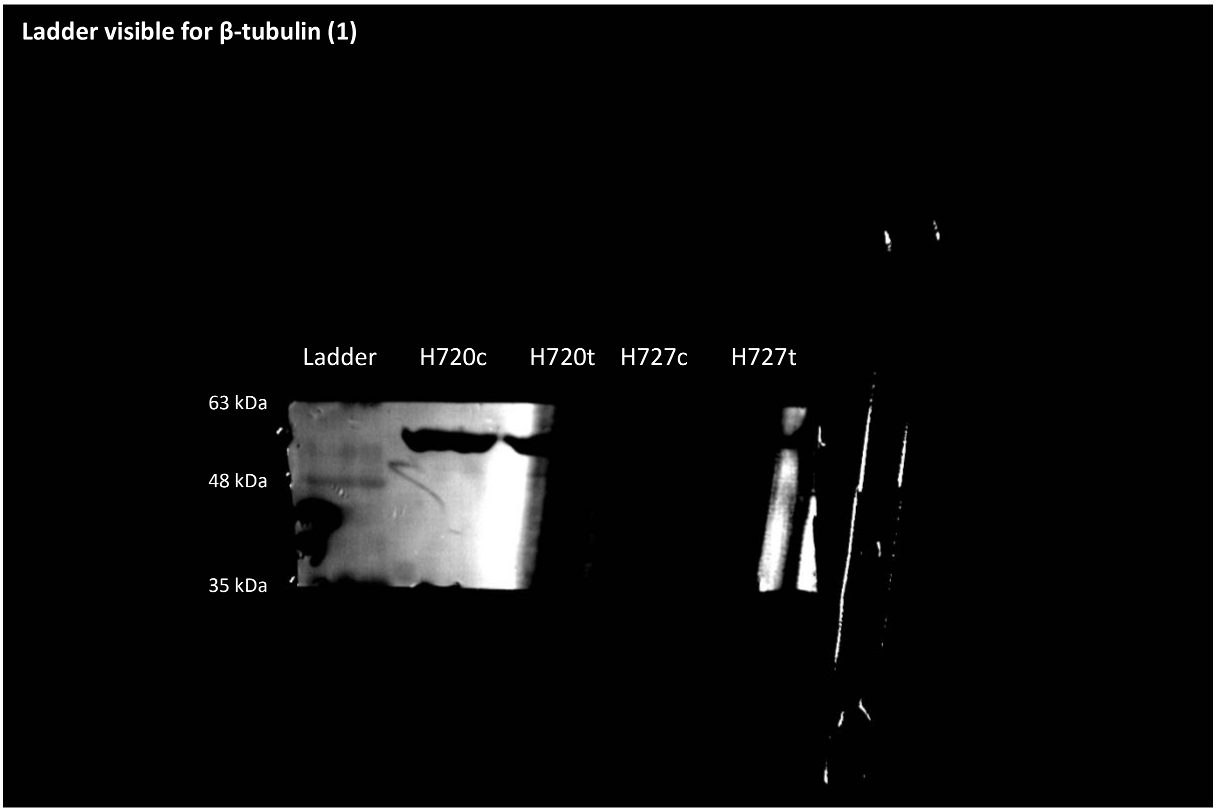
Ladder visible for β-tubulin (1)
Ladder H720c H720t H727c H727t
63 kDa
48 kDa
35 kDa

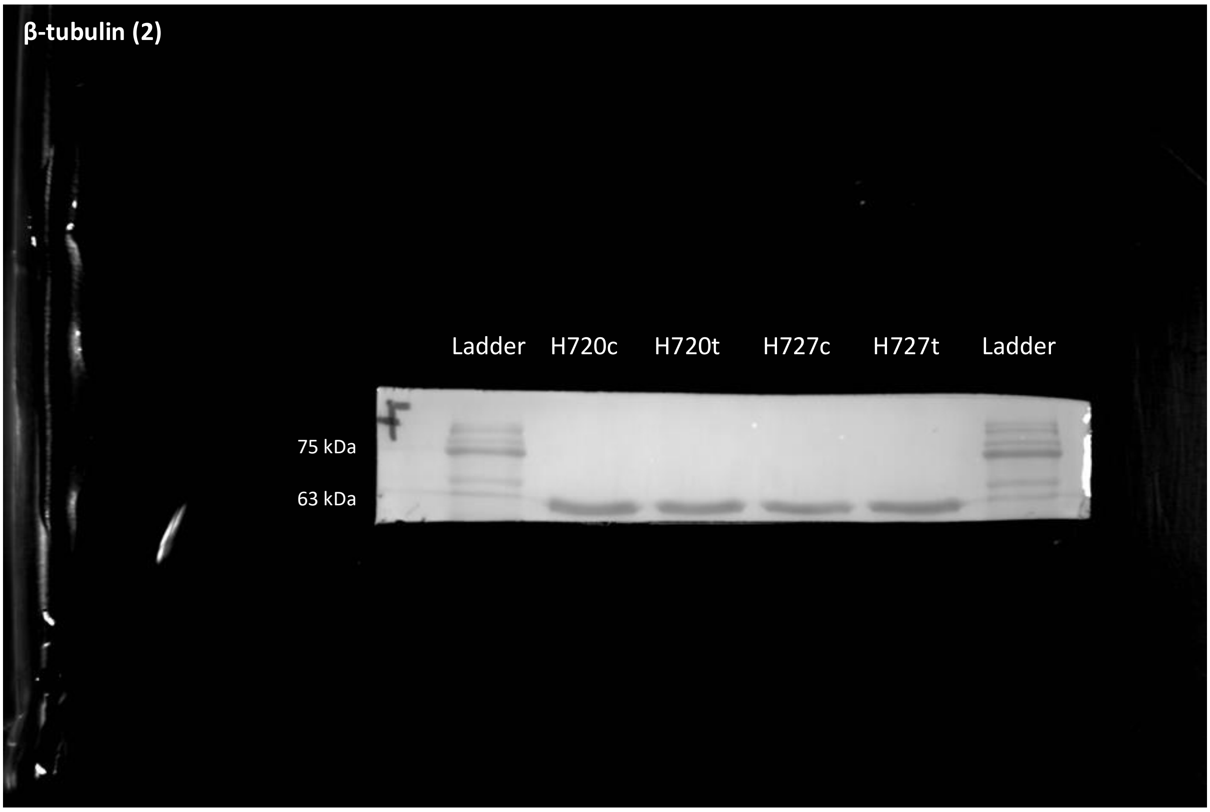

Supplementary Fig. 9. Uncropped images associated with Figure 7: detection of β-tubulin. Ladder shown separately once.
c = control
t = treated

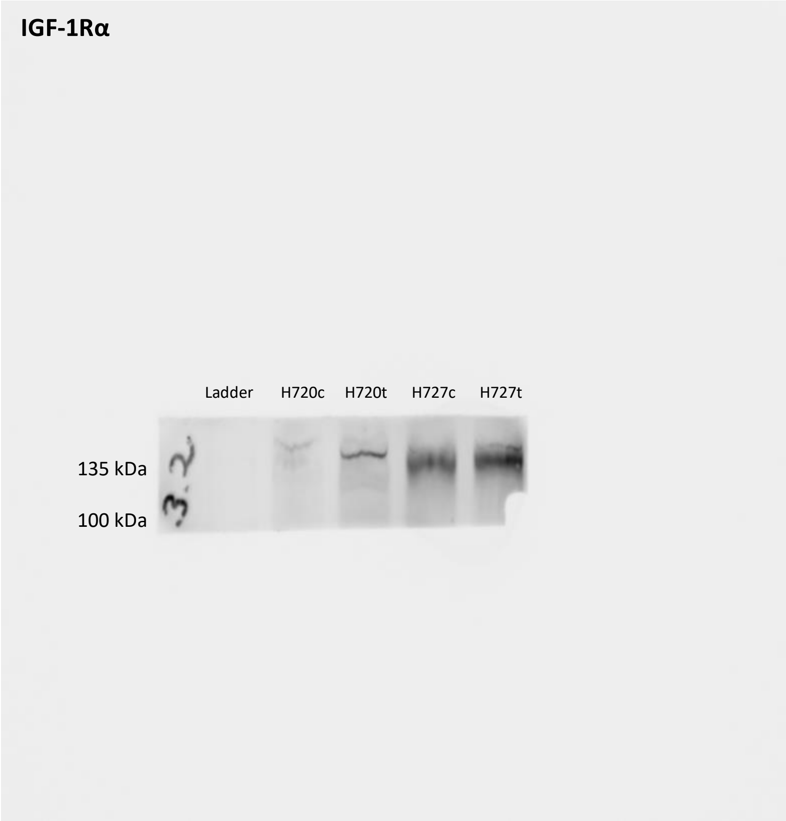

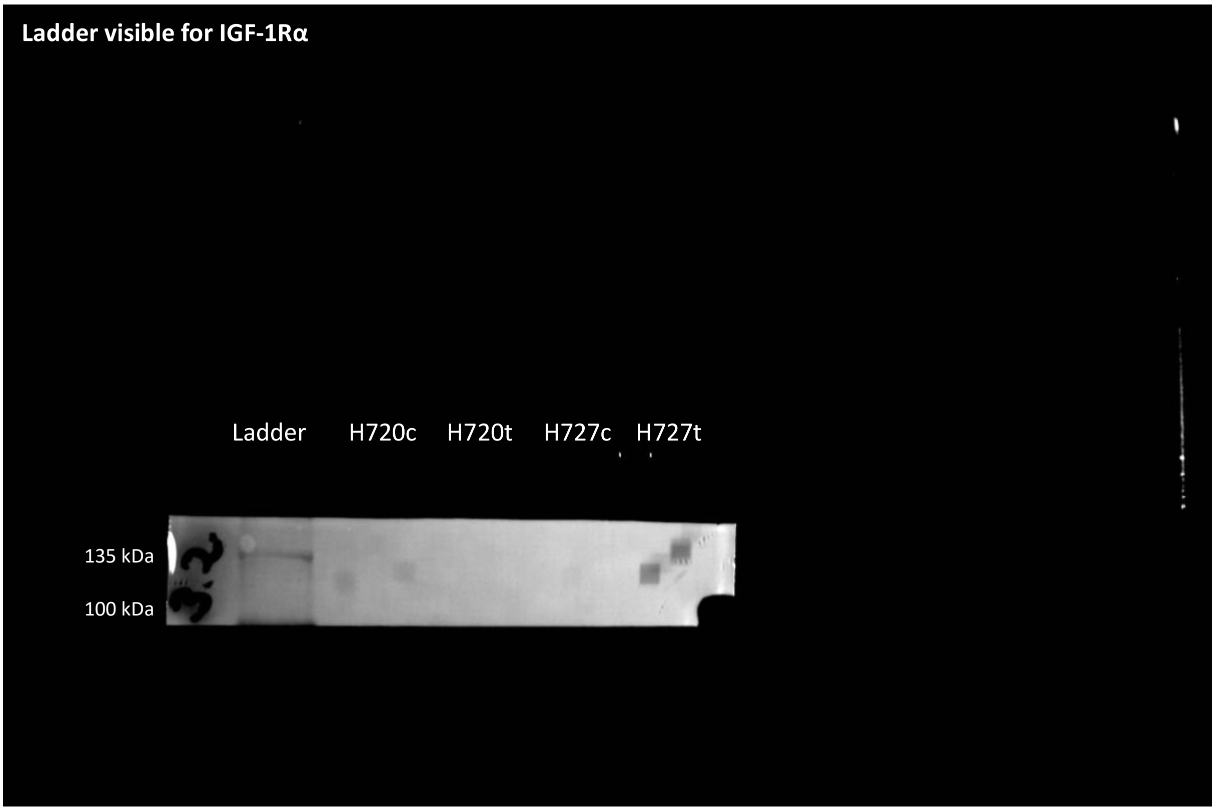

Supplementary Fig. 10. Uncropped image associated with Figure 7A: detection of IGF-1Rα. Ladder shown separately.
c = control
t = treated

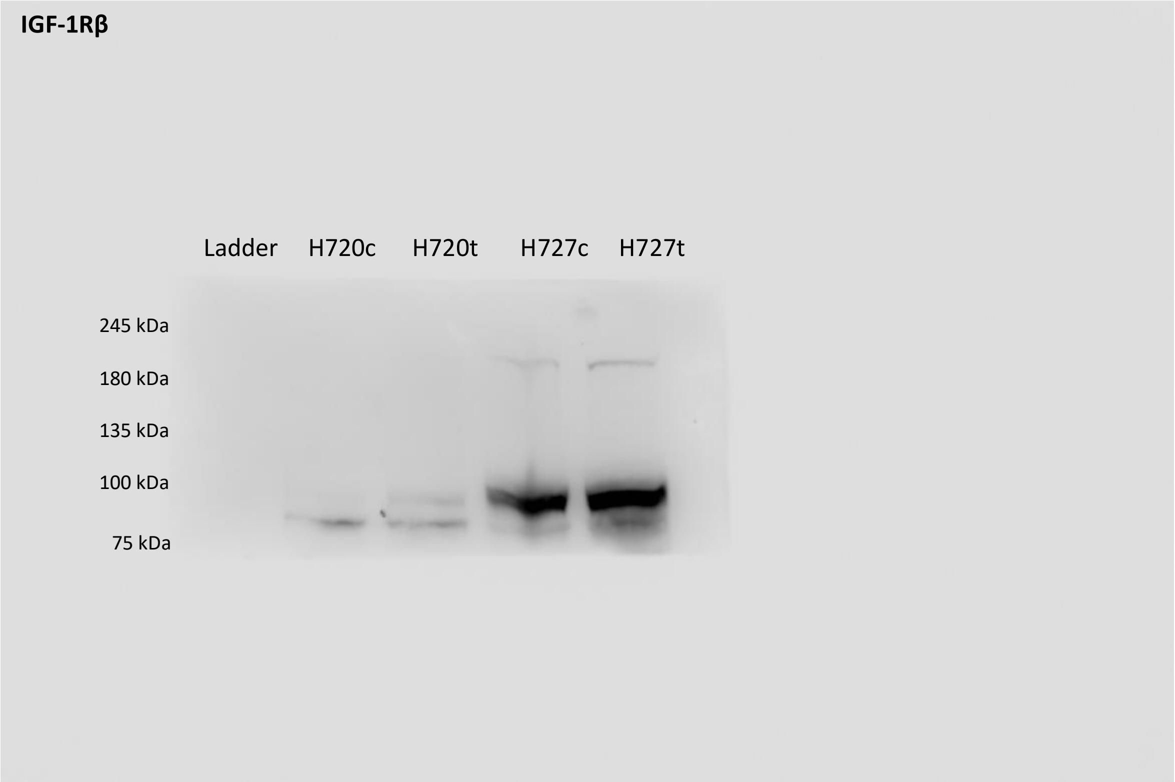

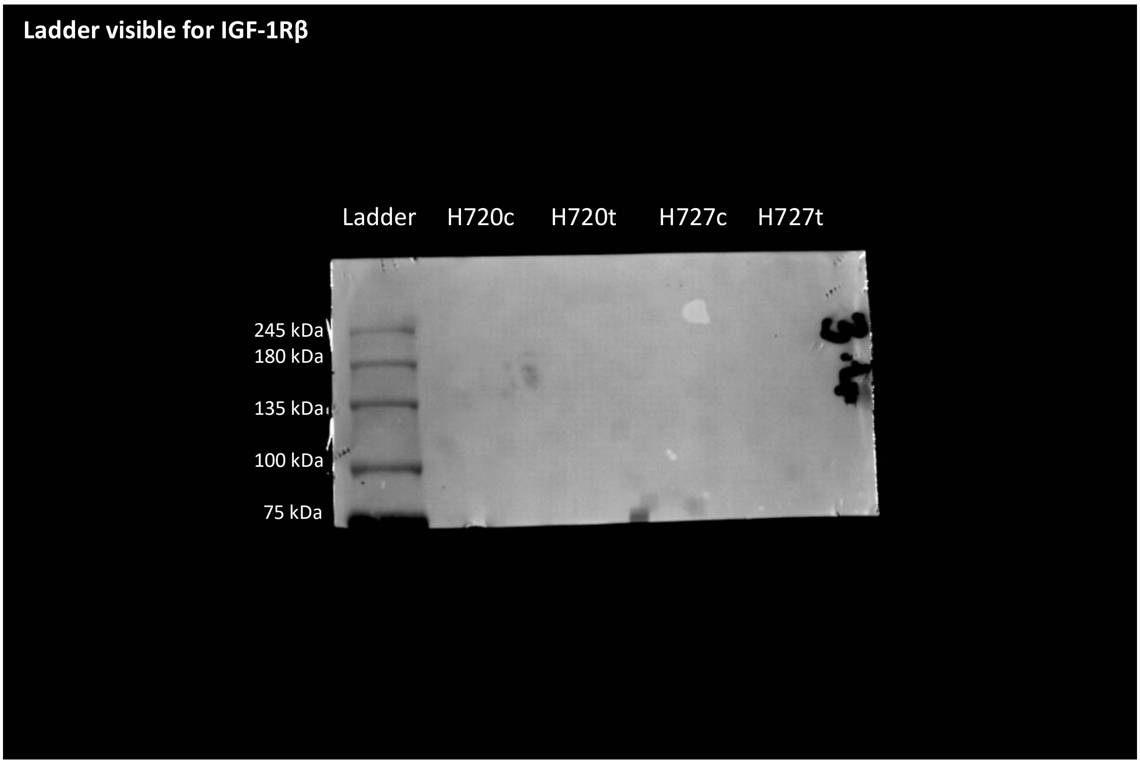

Supplementary Fig. 11. Uncropped image associated with Figure 7A: detection of IGF-1Rβ. Ladder shown separately.
c = control
t = treated

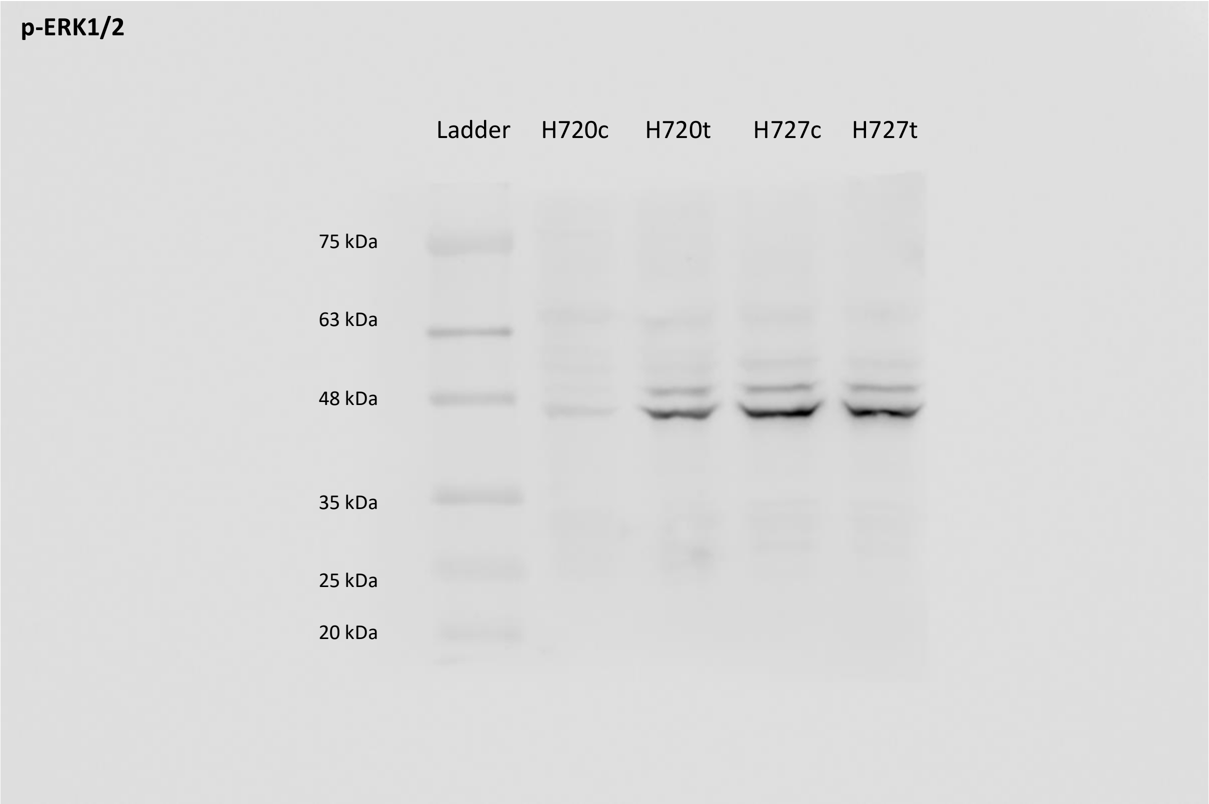

Supplementary Fig. 12. Uncropped image associated with Figure 7B: detection of p-ERK1/2.
c = control
t = treated

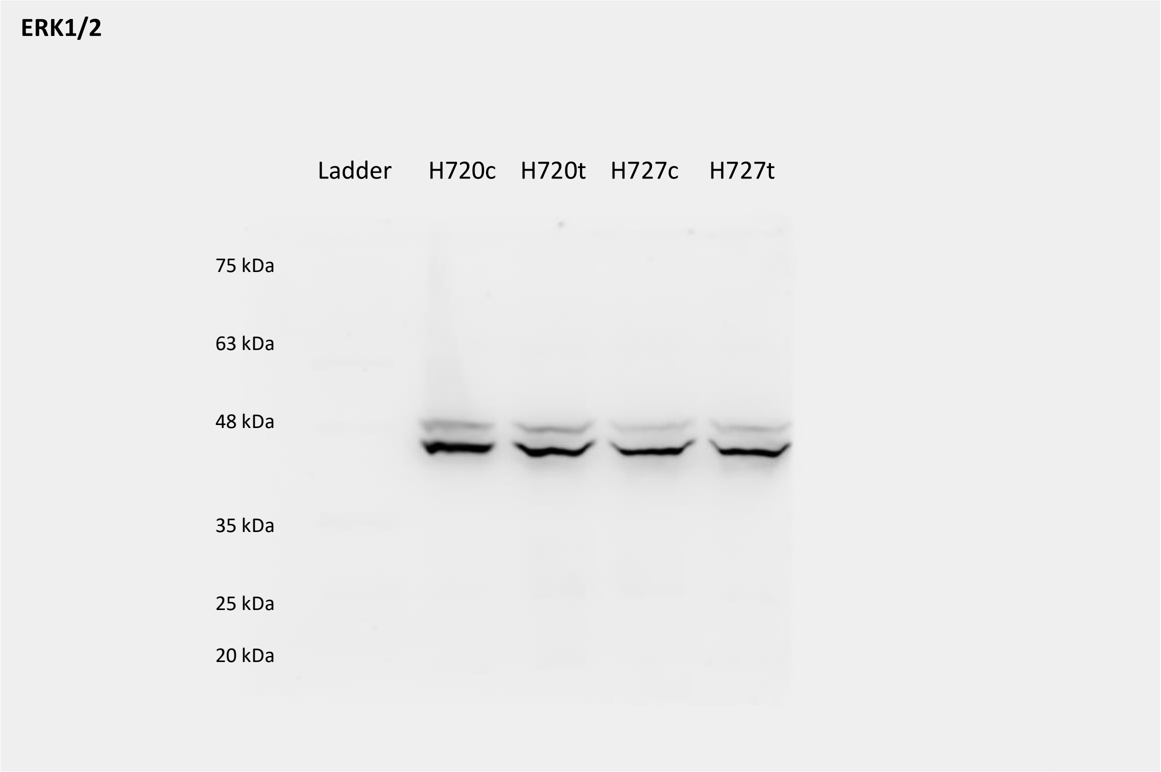

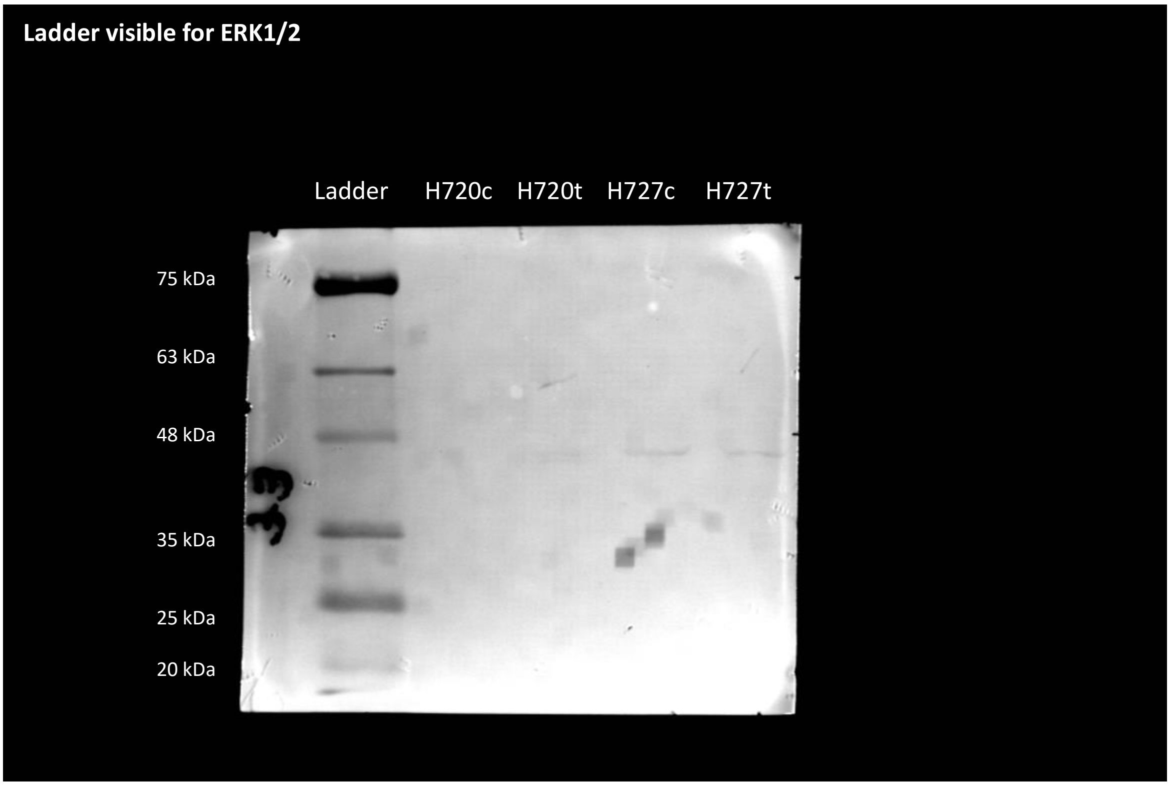

Supplementary Fig. 13. Uncropped image associated with Figure 7B: detection of ERK1/2. Ladder shown separately.
c = control
t = treated

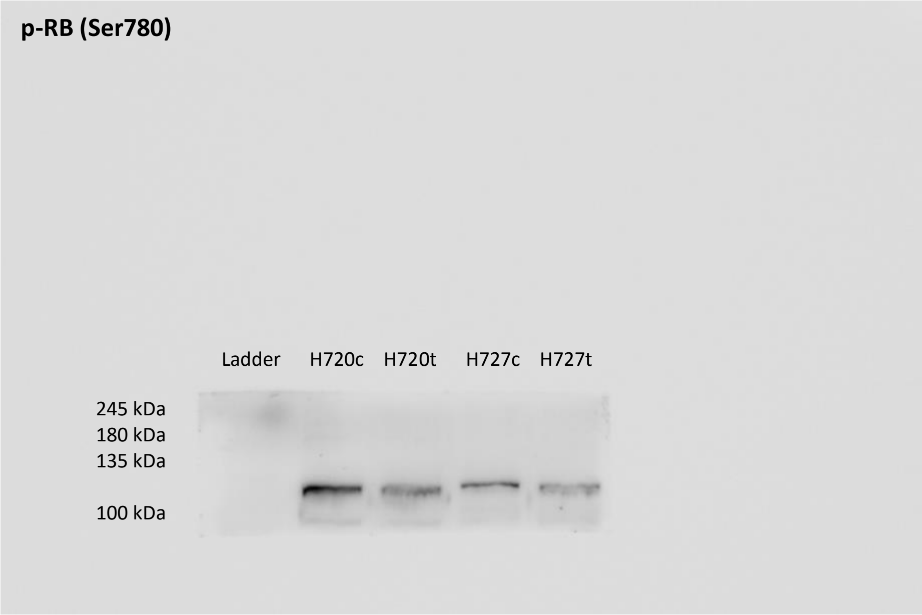

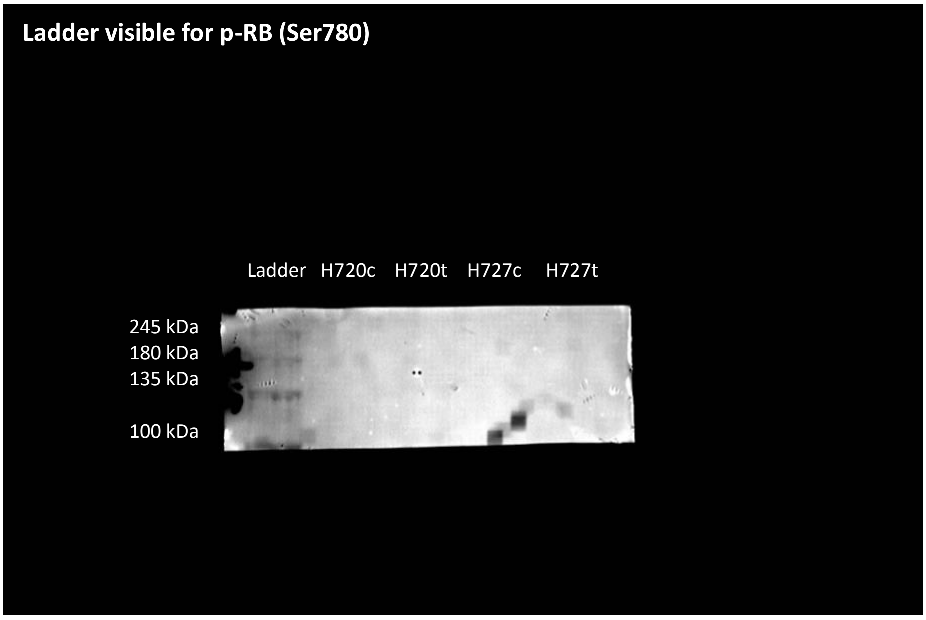

Supplementary Fig. 14. Uncropped image associated with Figure 7C: detection of p-RB (Ser780). Ladder shown separately.
c = control
t = treated

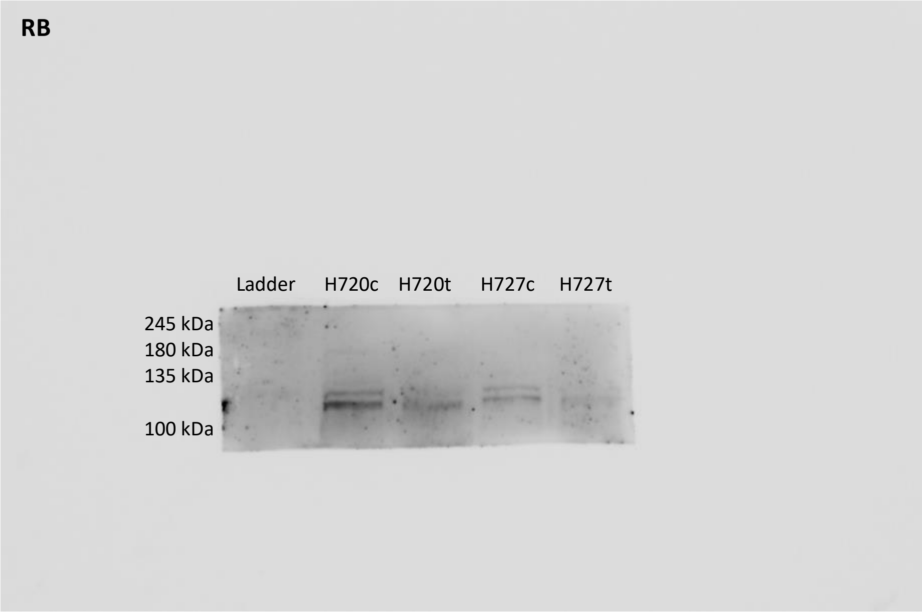

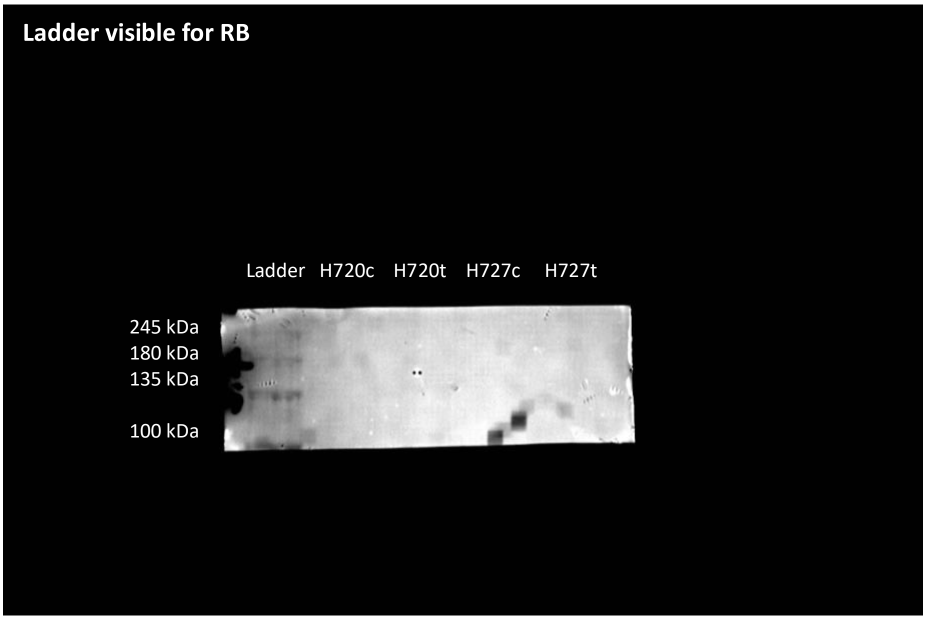

Supplementary Fig. 15. Uncropped image associated with Figure 7C: detection of RB. Ladder shown separately.
c = control
t = treated

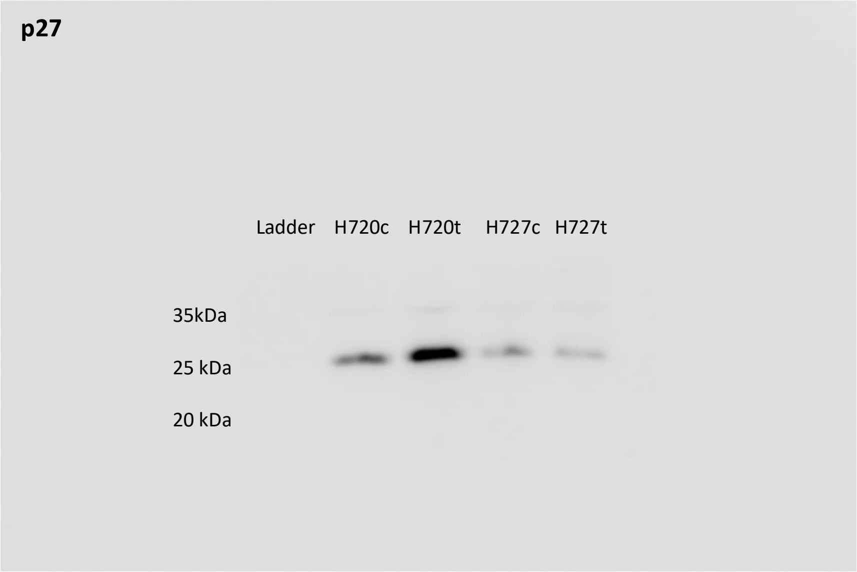

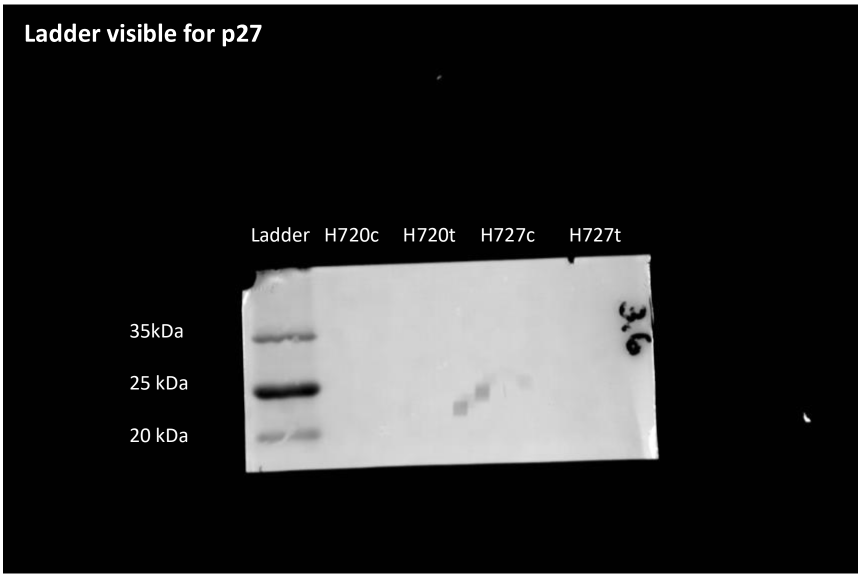

Supplementary Fig. 16. Uncropped image associated with Figure 7C: detection of p27.
Ladder shown separately.
c = control
t = treated

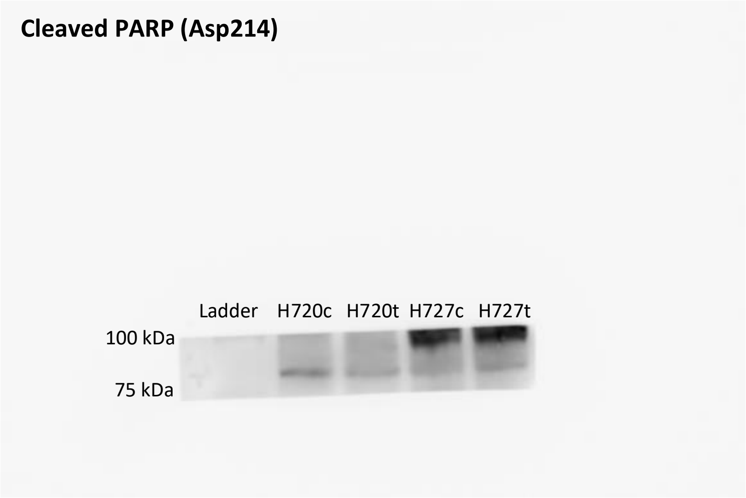

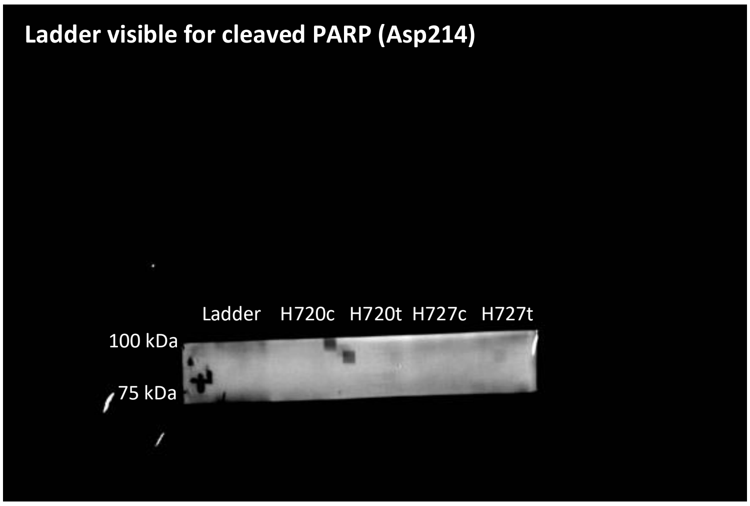

Supplementary Fig. 17. Uncropped image associated with Figure 7D: detection of cleaved PARP (Asp214). Ladder shown separately.
c = control
t = treated

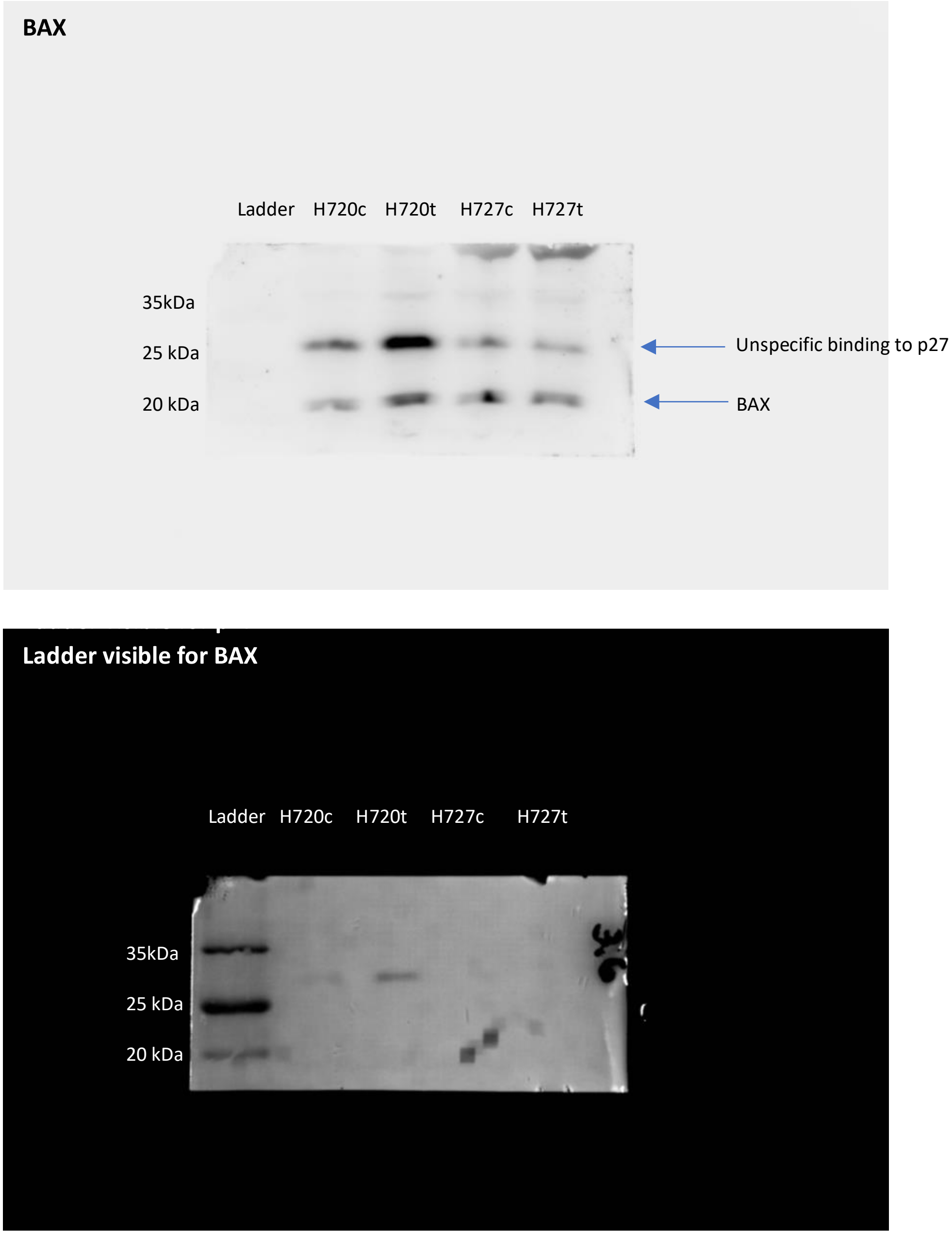

Supplementary Fig. 18. Uncropped image associated with Figure 7D: detection of BAX. Ladder shown separately.
c = control
t = treated
